# p66Shc signaling and autophagy impact on C2C12 myoblast differentiation during senescence

**DOI:** 10.1038/s41419-024-06582-0

**Published:** 2024-03-08

**Authors:** Yaiza Potes, Juan C. Bermejo-Millo, Catarina Mendes, José P. Castelão-Baptista, Andrea Díaz-Luis, Zulema Pérez-Martínez, Juan J. Solano, Vilma A. Sardão, Paulo J. Oliveira, Beatriz Caballero, Ana Coto-Montes, Ignacio Vega-Naredo

**Affiliations:** 1 Department of Morphology and Cell Biology, Faculty of Medicine, University of Oviedo, Oviedo, Spain; 2 Instituto de Investigación Sanitaria del Principado de Asturias (ISPA), Oviedo, Spain; 3 Institute of Neurosciences of the Principality of Asturias (INEUROPA), Oviedo, Spain; 4 CNC-UC, Center for Neuroscience and Cell Biology, University of Coimbra, Coimbra, Portugal; 5 CIBB, Center for Innovative Biomedicine and Biotechnology, University of Coimbra, Coimbra, Portugal; 6 PDBEB - Doctoral Program in Experimental Biology and Biomedicine, Institute of Interdisciplinary Research, University of Coimbra, Coimbra, Portugal; 7 Microbiology service, University Central Hospital of Asturias, Oviedo, Spain; 8 Geriatric Service, Monte Naranco Hospital, Av. Doctores Fernández Vega, Oviedo, Spain; 9 MIA-Portugal – Multidisciplinary Institute of Ageing, University of Coimbra, Coimbra, Portugal

**Keywords:** Differentiation, Ageing

## Abstract

During aging, muscle regenerative capacities decline, which is concomitant with the loss of satellite cells that enter in a state of irreversible senescence. However, what mechanisms are involved in myogenic senescence and differentiation are largely unknown. Here, we showed that early-passage or “young” C2C12 myoblasts activated the redox-sensitive p66Shc signaling pathway, exhibited a strong antioxidant protection and a bioenergetic profile relying predominantly on OXPHOS, responses that decrease progressively during differentiation. Furthermore, autophagy was increased in myotubes. Otherwise, late-passage or “senescent” myoblasts led to a highly metabolic profile, relying on both OXPHOS and glycolysis, that may be influenced by the loss of SQSTM1/p62 which tightly regulates the metabolic shift from aerobic glycolysis to OXPHOS. Furthermore, during differentiation of late-passage C2C12 cells, both p66Shc signaling and autophagy were impaired and this coincides with reduced myogenic capacity. Our findings recognized that the lack of p66Shc compromises the proliferation and the onset of the differentiation of C2C12 myoblasts. Moreover, the Atg7 silencing favored myoblasts growth, whereas interfered in the viability of differentiated myotubes. Then, our work demonstrates that the p66Shc signaling pathway, which highly influences cellular metabolic status and oxidative environment, is critical for the myogenic commitment and differentiation of C2C12 cells. Our findings also support that autophagy is essential for the metabolic switch observed during the differentiation of C2C12 myoblasts, confirming how its regulation determines cell fate. The regulatory roles of p66Shc and autophagy mechanisms on myogenesis require future attention as possible tools that could predict and measure the aging-related state of frailty and disability.

## Introduction

Age-related loss of skeletal muscle mass, strength and performance (sarcopenia) increases frailty and disability, leading to functional dependence. Age-induced muscle wasting is associated with a defective regenerative ability caused by a reduced resident adult muscle stem cells (satellite cells) number and function [1]. The multiple cellular divisions of skeletal muscle satellite cells throughout the lifespan progressively reduce telomere length, proliferation rate and differentiation potential, impacting on muscle regeneration in older individuals [2, 3].

Phosphoinositide 3-kinase/Akt/mammalian target of rapamycin signaling axis, which is tightly influenced by bioenergetic demands, is a critical pathway for cell growth and fate determination in stem cells [4]. Although energy metabolism is a major determinant of skeletal muscle mass maintenance [5], the metabolic remodeling and energy demands of satellite cells from quiescent to proliferative/non-differentiating, committed/differentiating and senescent states are poorly understood. Similarly to other stem cell subtypes [6, 7], satellite cells display reduced mitochondrial mass which is increased throughout differentiation, suggesting mitochondria as a potential regulator of metabolism during myogenesis [8, 9]. Moreover, mitochondrial metabolism is compromised with age, resulting in a shift in energy metabolism which anticipates the onset of sarcopenia [10]. Accordingly, dysfunctional mitochondria, an important source of reactive oxygen species (ROS), are considered primary initiators of myocyte atrophy during aging [11]. Given the postmitotic nature of muscle cells, the maintenance of cellular and energy homeostasis relies on the efficiency of quality control mechanisms [12]. Autophagy is a key component of the cellular quality control mechanisms that is tightly involved in balancing sources of energy during development [13, 14]. Moreover, it was proposed that dysfunctional mitochondria are selectively removed by autophagy due to increased free radical production, corroborating the redox control of autophagy [15]. Since mitochondrial dysfunction is implicated in aging and in several age-associated disorders [16–19], investigating mitochondrial function and cell quality control in muscle progenitor cells is important to develop novel pathways for therapeutic purposes and biomarkers discovery in sarcopenia.

The redox protein adapter p66Sch has a role in mitochondrial function [20]. p66Shc phosphorylation at Ser36 (pSer36-p66Shc) [21] is an important step in its translocation to mitochondria and/or mitochondria-associated membranes [22, 23] during aging or upon oxidative stress. pSer36-p66Shc triggers a cascade of events leading to an increase in free radicals production, which can contribute to mitochondrial damage under different conditions [22]. In fact, deletion of p66Shc decreases the incidence of aging-associated diseases and prolongs lifespan [24, 25]. p66Shc null mice, which have a longer lifespan, present abrogation of the p53 aging functions suggesting that p66Shc is a critical component of the aging pathway [26]. However, p66Shc signaling seems to be important due to the relevance of oxidative stress during myogenic differentiation [27]. Furthermore, p66Shc appears to regulate mitochondrial metabolism, since oxygen consumption was lower in p66Shc(-/-) mouse fibroblasts than in wild type cells [28]. Also, mitochondrial metabolism appears to regulate p66Sch activity [29]. A fraction of p66Shc within mitochondria forms a complex with HSP70, from where it is released after several types of stress [30]. During autophagy, p66Shc can be actively recruited to mitochondria undergoing metabolic or oxidative stress. Thus, quality control regulated by p66Shc seems to determine the fate of mitochondria and in turn the entire cell [31].

Growing evidence suggests that p66Shc activation is involved in deleterious processes and is critical in aging-related cellular alterations. However, the role of p66Shc and cell quality control by autophagy on myogenesis is unknown. The objective of the present work is to evaluate if the activation of p66Shc and the regulation of autophagy are critical points for muscle progenitor cells differentiation during their senescence. To explore this subject in a joint manner, we have studied the relevance of the redox-sensitive p66Shc signaling pathway and the cellular quality control by autophagy for mouse myoblastic C2C12 cells senescence and differentiation potential.

## Results

### Senescence of myogenic progenitors impedes myogenesis

**Fig. 1:**
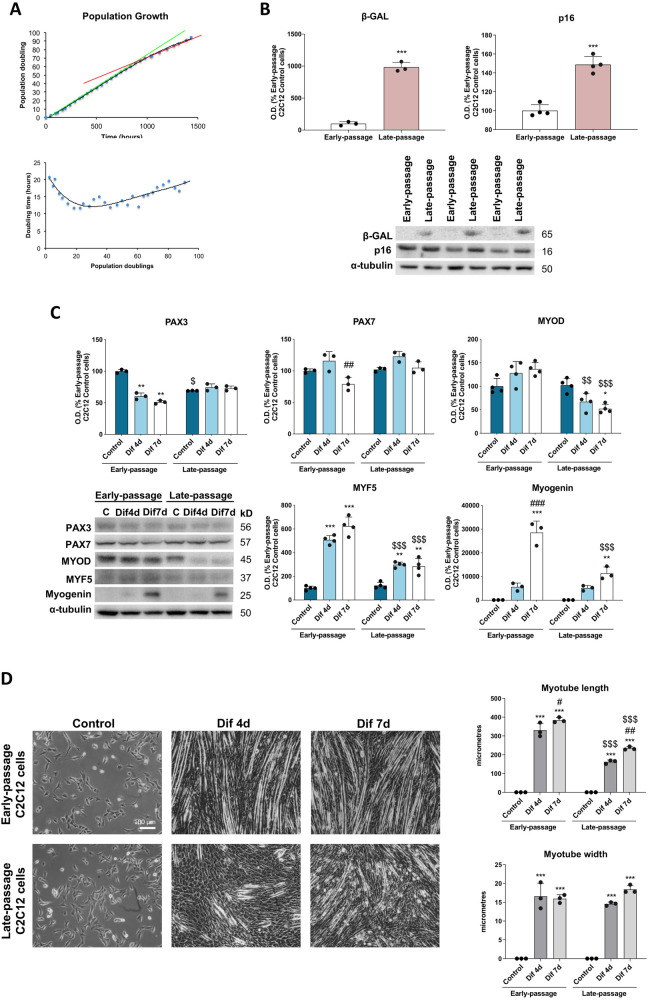
Replicative senescence alters C2C12 growth dynamics and myogenic differentiation. **A** Growth dynamics were evaluated by measuring population doublings at the end of every passage and the doubling population time. **B** Protein expression analysis of senescence markers (β-GAL and p16) (*n* = 4). Data are mean of optical density (O.D.) ± SD expressed as percentage of Early-passage C2C12 Control cells. α-tubulin was used as loading control. Statistical comparisons: *Early-passage vs. Late-passage. The number of symbols represents the level of statistical significance: one for *P* < 0.05, two for *P* < 0.01 and three for *P* < 0.001. **C** Protein expression analysis of myogenic markers (PAX3, PAX7, MYOD, MYF5 and myogenin) (*n* = 4). Data are mean of optical density (O.D.) ± SD expressed as percentage of Early-passage C2C12 Control cells. α-tubulin was used as loading control. **D** Phase contrast light microscopy of proliferating early- and late-passage C2C12 myoblasts and differentiating myotubes on day 4 (Dif4d) and day 7 (Dif7d) and myotube length and width quantification) (*n* = 3). Scale bar represents 100 μm. Statistical comparisons: *Control vs. Differentiation; # Dif4d vs. Dif7d; $ Early-passage vs. Late-passage. The number of symbols represents the level of statistical significance: one for *P* < 0.05, two for *P* < 0.01 and three for *P* < 0.001.

The multiple population doubling model allows to investigate in vitro the multiple divisions that occur in vivo, in muscle stem cells, with ageing. To monitor C2C12 growth rates and determine its replicative senescence state, during cell proliferation the population doubling (PD) and doubling time (DT) of each passage were calculated. Initially, during the first 8 PDs the DT varied between 20 and 15 h until cells adapted to the growing conditions. Afterwards, the DT of C2C12 cells was approximately 12 h and remained stable up to 65 PDs. From this point, the DT increased gradually until reaching values between 18 and 20 h, which was associated with a decline in proliferative potential due to the cell-cycle arrest (Fig. 1A). Therefore, 65 PDs can be established as the cutoff point for the replicative senescence of C2C12 cell line. Additionally, to confirm cellular senescence, the protein expression of β-galactosidase (β-GAL) and p16 were assessed in early and late-passage C2C12 cells. Accordingly, late-passage C2C12 cells, with PDs higher than 65, exhibited significantly higher levels of both senescence markers (Fig. 1B).

Given that senescence antagonizes myogenesis [32], to further characterize the senescent condition of C2C12 myoblasts, we studied the differentiation potential of early-passage or “young” and late-passage or “senescent” C2C12 cells by analyzing the expression of the upstream regulators of myogenesis (paired box protein 3 (PAX3) and 7 (PAX7)) and the early (myogenic differentiation 1 (MYOD) and myogenic factor 5 (MYF5) and late (myogenin) skeletal muscle differentiation markers. Despite late-passage C2C12 myoblasts, compared to early-passage myoblasts, only showed major changes in PAX3 protein content which experienced a 30% reduction, cellular senescence had a significant effect on the extent of differentiation. Early-passage C2C12 cells exhibited a progressive 50 and 21% decline of PAX3 and PAX7 protein markers, respectively, along differentiation, which was accompanied by a sharp increase in the protein levels of MYF5 and myogenin. MYOD protein levels persisted unaltered. However, differentiated late-passage C2C12 cells experienced no changes in PAX3 expression, a 61% decline of MYOD expression, together with a small increase of MYF5 and myogenin thorough differentiation. Moreover, differentiated late-passage C2C12 cells exhibited a 69 and 60% reduction in MYF5 and myogenin levels in comparison to differentiated early-passage cells (Fig. 1C). Images after 4 and 7 days of differentiation (Dif4d and Dif7d, respectively) of early-and late-passage C2C12 cells obtained with phase contrast light microscope evidenced a reduced number and myotube length of late-passage C2C12 myotubes. No differences in the myotube width were observed (Fig. 1D). Thus, cell senescence impairs the ability of C2C12 cells to differentiate into myotubes.

### Senescence leads to a metabolic reprogramming of C2C12 myoblasts and myotubes

**Fig. 2:**
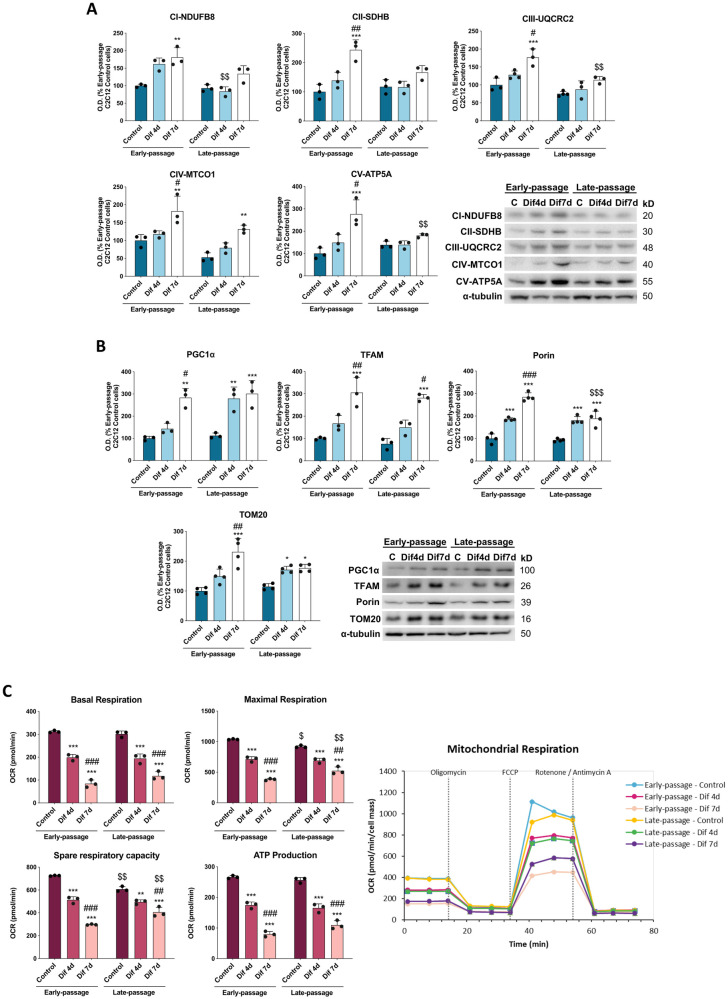
Senescence remodels OXPHOS complexes and mitochondrial respiration. **A** Protein expression analysis of OXPHOS subunits from each complex (NFUFB8, SDHB, UQCRC2, MTCO1, ATP5A) (*n* = 3). Data are mean of optical density (O.D.) ± SD expressed as percentage of Early-passage C2C12 Control cells. α-tubulin was used as loading control. **B** Protein expression analysis of the mitochondrial biogenesis markers PGC1α and TFAM and the mitochondrial mass markers Porin and TOM20) (*n* = 3). Data are mean of optical density (O.D.) ± SD expressed as percentage of Early-passage C2C12 Control cells. α-tubulin was used as loading control. **C** Mitochondrial bioenergetics evaluation by measuring basal respiration, maximal respiration, spare respiratory capacity and respiration associated to ATP production, following addition of oligomycin, FCCP and rotenone/antimycin A (*n* = 3). Data are mean ± SD. Statistical comparisons: *Control vs. Differentiation; # Dif4d vs. Dif7d; $ Early-passage vs. Late-passage. The number of symbols represents the level of statistical significance: one for *P* < 0.05, two for *P* < 0.01 and three for *P* < 0.001.

Since energy metabolism is implicated in cell growth and fate determination [33], we measured bioenergetic parameters in early- and late-passage C2C12 myoblasts and during their differentiation into myotubes. To characterize mitochondrial function, we first measured the levels of some subunits of the mitochondrial electron transport chain (ETC) complexes, namely NADH dehydrogenase (ubiquinone) 1 β subcomplex 8 (NDUFB8) from complex I; succinate dehydrogenase (ubiquinone) iron-sulfur subunit (SDHB) from complex II; ubiquinol-cytochrome c reductase core protein II (UQCRC2) from complex III; cytochrome c oxidase subunit I (MTCO1) from complex IV and ATP synthase subunit α (ATP5A) from complex V by immunodetection. Even though no significant alterations were observed between early- and late-passage C2C12 myoblasts, senescence-induced differences during the differentiation process. Our results showed an increased expression of all the subunits throughout the differentiation process in early-passage C2C12 cells, being significantly higher in Dif7d C2C12 cells. This was also accompanied by a progressive increase of the mitochondrial biogenesis markers PGC1α and TFAM, as well as the mitochondrial porin/VDAC1 and TOM20 content. Although late-passage cells also exhibited slightly increased protein expression of all the subunits of ETC complexes, mitochondrial biogenesis markers, and the porin/VDAC1 and TOM2O throughout differentiation process, differentiated late-passage C2C12 cells presented reduced expression of subunits from complexes I, III and V, and no major expression variations of SDHB and MTCO1 in comparison with differentiated early-passage C2C12 cells. Additionally, the mitochondrial biogenesis markers PGC1α and TFAM also remained unaltered. However, porin and TOM20 expression was lower in late-passage Dif7d C2C12 cells (Fig. 2A, B).

To evaluate if this ETC remodeling in terms of protein content affects mitochondrial function, we measured cellular oxygen consumption rate (OCR) using the Seahorse XFe96 extracellular flux analyzer. Although basal respiration and ATP levels remained unaltered between early- and late-passage C2C12 myoblasts, it was found a 12% decline of maximal respiration and 16% decline of spare respiratory capacity (the difference between the maximal respiration and basal respiration) in late-passage C2C12 myoblasts when compared to early-passage cells. During the differentiation of early-passage C2C12 cells, despite early-passage myotubes exhibited higher levels of subunits from ETC complexes than early-passage C2C12 myoblasts, it was observed decreases in basal respiration by 73%, in maximal mitochondrial respiration by 63%, and in spare respiratory capacity by 59%. Accordingly, ATP production-related OCR in early-passage C2C12 cells also decreased after their differentiation. Surprisingly, although differentiation of late-passage C2C12 cells also reduces mitochondrial respiration rates, the maximal mitochondrial respiration and the spare respiratory capacity were 11 and 16% higher in late-passage Dif7d cells than in early-passage Dif7d cells. However, the ATP production-related OCR was not significantly higher (Fig. 2C).

**Fig. 3:**
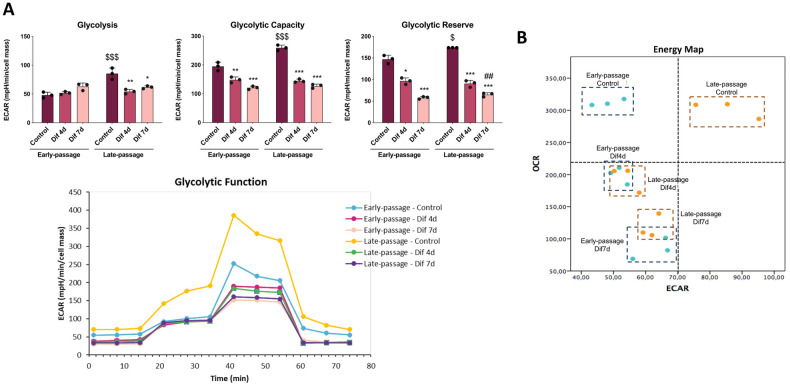
Senescence promotes glycolytic metabolism. **A** Extracellular acidification rates indirectly representing glycolysis, glycolytic capacity and glycolytic reserve based on the responses to glucose, oligomycin and 2-deoxy-d-glycose. Data are mean ± SD. **B** Energy map showing the correlation between oxygen consumption rate (OCR) and extracellular acidification rate (ECAR) (*n* = 3). Statistical comparisons: *Control vs. Differentiation; # Dif4d vs. Dif7d; $ Early-passage vs. Late-passage. The number of symbols represents the level of statistical significance: one for *P* < 0.05, two for *P* < 0.01 and three for *P* < 0.001.

Next, to further understand the metabolic profiling we evaluated glycolysis by measuring extracellular acidification rate (ECAR) using the Seahorse XFe96 analyzer. Late-passage control C2C12 cells exhibited higher glycolysis, glycolytic capacity and glycolic reserve (the difference between the glycolytic capacity and glycolysis) than early-passage control cells. Early-passage control, Dif4d and Dif7d C2C12 cells showed a similar glycolytic response. However, declines in glycolytic capacity and in glycolytic reserve were found when early-passage C2C12 cells were differentiated. Glycolysis, glycolytic capacity and glycolytic reserve of late-passage C2C12 cells suffered a reduction after 4 days of differentiation, showing a glycolytic response similar to differentiated early-passage C2C12 cells (Fig. 3A).

The OCR/ECAR ratio was calculated to evaluate the cellular preference for OXPHOS versus glycolysis. Notably, the OCR/ECAR ratio revealed that early-passage control C2C12 cells are heavily reliant on mitochondrial activity for energy production, while in contrast, late-passage control C2C12 cells exhibited higher metabolic phenotype characterized by a high reliance on both mitochondrial activity and glycolysis. Moreover, early- and late-passage C2C12 cells showed a progressive shift from a higher to a lower metabolic phenotype during differentiation process. Both early- and late-passage C2C12 cells showed a gradual reduction of the utilization of OXPHOS for ATP production upon differentiation, which was accompanied by decreased acidification in late-passage cells (Fig. 3B). Altogether, our findings indicate that senescent C2C12 myoblasts undergo a switch in energy metabolism, increasing the metabolic activities for ATP production that progressively decline during their differentiation.

### Senescence alters the oxidative environment and attenuates p66Shc activation in C2C12 myoblasts

**Fig. 4:**
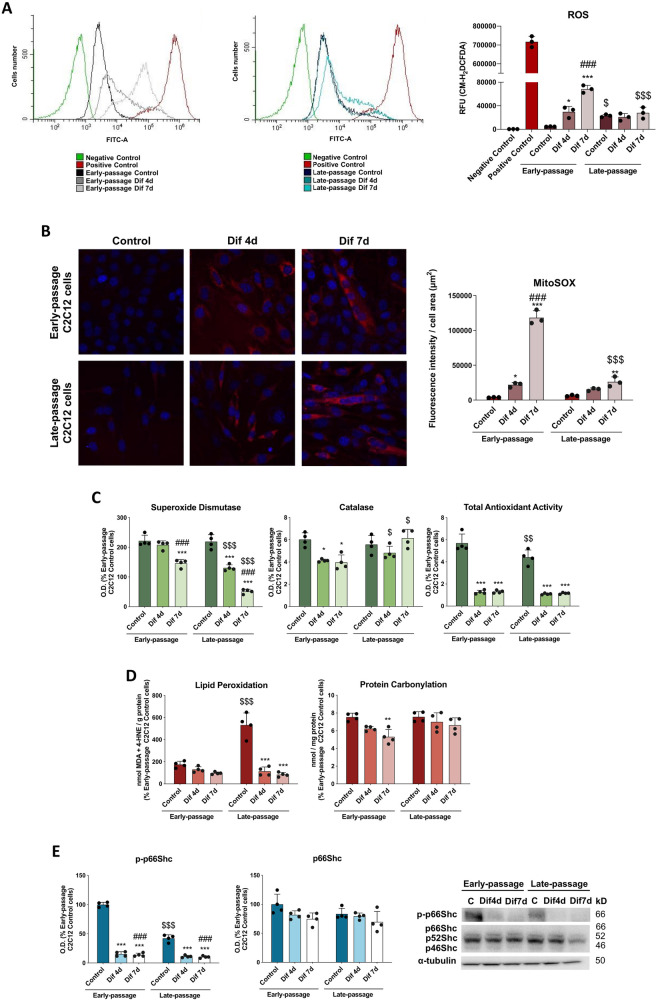
Senescence represses p66Shc activation and alters oxidative stress status in C2C12 cells. **A** Determination of reactive oxygen species (ROS) levels by flow cytometric analysis of CM-H_2_DCFDA staining (*n* = 3). Data are mean of relative fluorescence unit (RFU) ± SD. **B** The fluorogenic substrate MitoSOX was used to study mitochondrial-derived ROS production by confocal microscopy analysis (*n* = 3). Data are mean of fluorescence intensity ± SD. **C** Antioxidant system evaluation by the activity determination of superoxide dismutase and catalase enzymes and the total antioxidant activity (*n* = 4). The inhibition of hematoxylin autoxidation to hematin was assessed to determine SOD activity (*n* = 4). Catalase activity was assayed by measuring H_2_O_2_ conversion in O_2_ and H_2_O (*n* = 4). Total antioxidant activity was analyzed using ABTS/H_2_O_2_/HRP method (*n* = 4). **D** Levels of lipid peroxidation and protein carbonylation were also analyzed. MDA and 4-HNE end-products were measured to determine lipid peroxidation levels (*n* = 4). Protein carbonylation was evaluated by measuring the reaction of 2,4-dinitrophenylhydrazine with the carbonyl groups of damaged proteins (*n* = 4). Data are mean ± SD. **E** Protein expression analysis of the Shc-transforming protein 1 p66Shc and p-p66Shc (*n* = 4). Data are mean of optical density (O.D.) ± SD expressed as percentage of Early-passage C2C12 Control cells. α-tubulin was used as loading control. Statistical comparisons: *Control vs. Differentiation; # Dif4d vs. Dif7d; $ Early-passage vs. Late-passage. The number of symbols represents the level of statistical significance: one for *P* < 0.05, two for *P* < 0.01 and three for *P* < 0.001.

Since energy metabolism is linked to the production of ROS, we next investigated whether cell senescence and cell differentiation of C2C12 myoblasts impact on oxidative stress parameters. Late-passage control C2C12 cells exhibited significantly higher levels of total cellular ROS and a slight increase in mitochondrial-specific ROS than early-passage control C2C12 cells. Although the activities of the antioxidant enzymes superoxide dismutase (SOD) and catalase (CAT) were unaltered, the total antioxidant activity (TAA) was significantly lower in late-passage myoblasts indicating that senescent myoblasts are not only characterized by increased production of ROS but also by reduced antioxidant defense system. As a result of this redox imbalance, increased levels of lipid peroxidation (LPO) were observed in late-passage C2C12 myoblasts. However, there were not found differences in protein carbonylation levels between early- and late-passage C2C12 myoblasts. Differentiation of early-passage C2C12 cells was accompanied by a progressive increase in total cellular ROS production and mitochondrial-specific ROS levels, together with a decline in antioxidant activities, without impacting on LPO levels but decreasing protein carbonylation. However, the differentiation protocol in late-passage C2C12 cells was not able to induce this total cellular ROS burst. Similarly, mitochondrial-specific ROS levels were not markedly increased during differentiation of late-passage C2C12 cells as in early-passage counterparts. In addition, late-passage C2C12 myotubes exhibited an altered antioxidant defense evidenced by reduced SOD response and unaltered CAT activity. Interestingly, LPO levels were decreased during differentiation of late-passage C2C12 cells (Fig. 4A–D).

Considering that the p66 isoform of Src homologous-collagen homologue adaptor 1 (SHC) plays a pivotal role in energy metabolism regulation and mitochondrial ROS production [34], we next investigated the activation of this signaling pathway during the C2C12 differentiation into myotubes. Early- and late-passage C2C12 myoblasts showed higher phosphorylation of p66Shc at serine 36 when compared with the differentiated counterparts suggesting a possible role of this redox-sensitive signaling pathway on myoblasts survival, maintenance, and differentiation. However, it is noteworthy that pSer36-p66Shc protein levels in late-passage C2C12 myoblasts were significantly lower than in early-passage myoblasts (Fig. 4E). The total protein expression of the SHC1 isoforms (p66Shc, p52Shc and p46Shc) persisted unaltered in all the experimental groups (Supplementary Fig. 1).

Importantly, we demonstrate here that senescence of C2C12 cells reduces p66Shc activation but promotes oxidative damage. Additionally, senescent C2C12 myotubes do not trigger total cellular ROS and mitochondrial-specific ROS production, which is necessary to direct the differentiation of C2C12 myoblasts.

### p66Shc depletion alters myogenic commitment and differentiation in senescent C2C12 cells

**Fig. 5:**
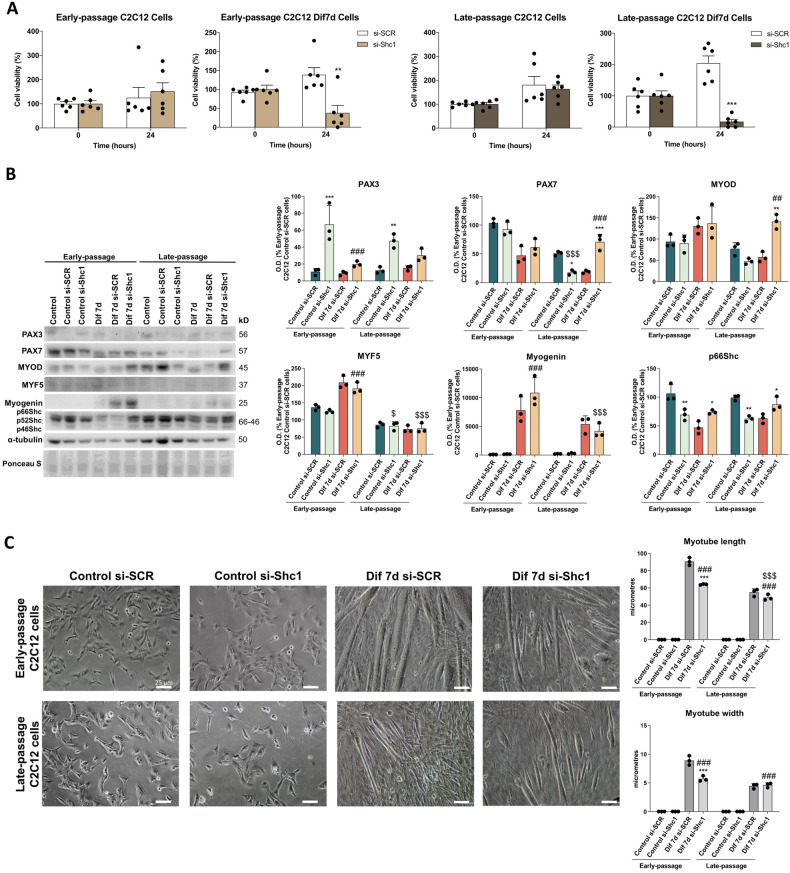
Silencing the adaptor gene Shc1 alters C2C12 cell commitment and differentiation. **A** Effect of Shc1 silencing (si-Shc1) on cell viability of Early-passage and Late-passage Control C2C12 and Dif7d C2C12 cells after 0- and 24-h treatment (*n* = 6). Data are mean ± SD. Statistical comparisons: *si-SCR vs. si-Shc1. The number of symbols represents the level of statistical significance: one for *P* < 0.05, two for *P* < 0.01 and three for *P* < 0.001. **B** Protein expression analysis of myogenic markers (PAX3, PAX7, MYOD, MYF5 and myogenin) and p66Shc levels in Early- and Late-passage Control C2C12 myoblasts and differentiating myotubes on day 7 (Dif7d) transfected with Shc1 (si-Shc1) or with scrambled siRNA (si-SCR) (*n* = 3). Data are mean of optical density (O.D.) ± SD expressed as percentage of Early-passage Control si-SCR C2C12 cells. Ponceau staining was used as loading control. **C** Phase contrast light microscopy of proliferating Early-passage and Late-passage Control si-SCR and si-Shc1 cells and differentiating myotubes Dif7d si-SCR and Dif7d si-Atg7 and myotube length and width quantification (*n* = 3). Scale bar represents 100 μm. Statistical comparisons: *si-SCR vs. si-Shc1; # Control vs. Dif7d; $ Early-passage vs. Late-passage. The number of symbols represents the level of statistical significance: one for *P* < 0.05, two for *P* < 0.01 and three for *P* < 0.001.

To further understand the role of p66Shc signaling during C2C12 myoblasts differentiation and senescence, we evaluated the cell viability/proliferation after silencing by Shc1 siRNA (si-Shc1) transfection. First, we determined the transfection efficiency of siRNA-mediated depletion of SHC1, that was found to be around 82% in early-passage C2C12 myoblasts and 59% in late-passage C2C12 myoblasts after 24 h. After 48 h, siRNA transfection efficiency was reduced about 42 and 22% in early- and late-passage C2C12 myoblasts, respectively (Supplementary Fig. 2). Our study showed that the loss of Shc1 did not induce any alteration on cell viability after 24 of transfection in early- and late-passage C2C12 myoblasts. However, Shc1 silencing reduced the viability of early- and late-passage Dif7d C2C12 cells at 24 h, evidencing an important role of this protein for the differentiation process (Fig. 5A).

To evaluate whether p66Shc signaling may impact on directing proper differentiation of C2C12 cells, we next studied the effect of Shc1 silencing on the expression of myogenic differentiation markers. Shc1 silencing in early-passage control C2C12 myoblasts only increased PAX3 protein expression levels. Curiously, Shc1 silencing in late-passage C2C12 myoblasts also induced high levels of PAX3 but decreasing PAX7 content, a phenotype associated with enhanced ability of long-term proliferation and slower differentiation capacity [35]. No major changes in the myogenic markers were found after the differentiation of early-passage si-Shc1 cells, whereas late-passage Dif7d si-Shc1 cells exhibited increased PAX7 and MYOD protein expression levels (Fig. 5B), which is associated to asynchronously proliferating conditions [36]. Phase contrast images of si-Shc1 silenced cells showed lower myoblast aling and fusion while non-silenced myotubes showed a more developed and multinucleated shape. Silenced late-passage myotubes exhibited significantly reduced length in comparison to their early-passage counterparts (Fig. 5C). Overall, our results indicate that the lack of p66Shc alters C2C12 cellular commitment and myogenic differentiation.

### Senescence diminishes the autophagic flux in C2C12 myoblasts but increases autophagic response during the differentiation process

**Fig. 6:**
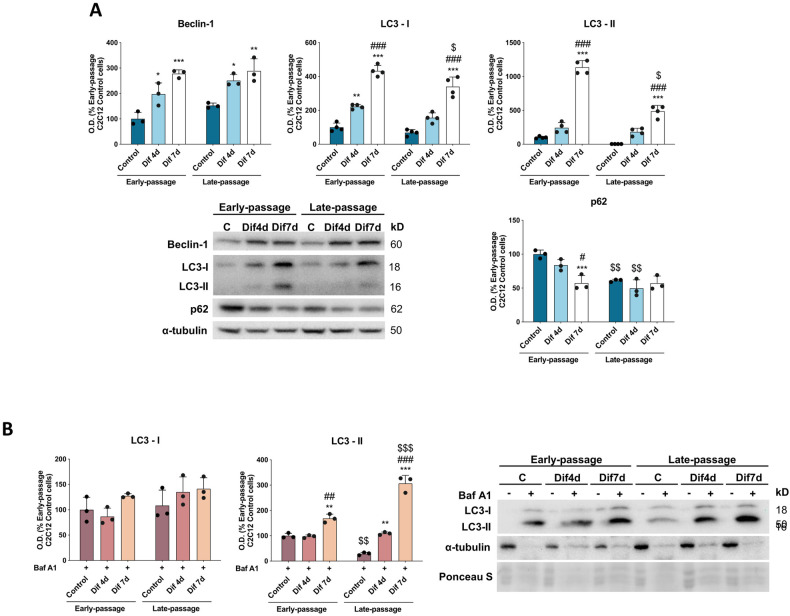
Senescence alters autophagic flux. **A** Protein expression analysis of autophagy markers (Beclin-1, LC3-I, LC3-II and SQSTM1/p62) (*n* = 3). **B** Detection of autophagic flux after inhibiting autophagosome-lysosome fusion with bafilomycin A1 by the protein expression analysis of LC3-I and LC3-II (*n* = 3). Data are mean of optical density (O.D.) ± SD expressed as percentage of Early-passage C2C12 Control cells. Ponceau staining was used as loading control. Statistical comparisons: *Control vs. Differentiation; # Dif4d vs. Dif7d; $ Early- vs. Late-passage. The number of symbols represents the level of statistical significance: one for *P* < 0.05, two for *P* < 0.01 and three for *P* < 0.001.

Macroautophagy constitutes an intracellular quality control mechanism through lysosome-dependent degradation, considered to be active in development and differentiation [37]. Thus, to evaluate the role of autophagy in C2C12 myoblast maintenance and differentiation, we measured the protein levels of Beclin-1, which is a class III phosphatidylinositol 2-kinase interacting protein with a role as a central regulator of autophagy in mammalian cells; the conversion of microtubule-associated protein 1 light chain 3 (LC3-I) to the phosphatidylethanolamine-conjugated form (LC3-II), a marker of autophagosomes; and sequestosome-1 (SQSTM1/p62), a multifunctional protein that can be used as marker of autophagic flux. Although our results showed no significant differences in Beclin-1 and LC3-II protein content between early- and late-passage C2C12 cells, SQSTM1/p62 protein levels were significantly lower in late-passage myoblasts suggesting increased autophagic degradation (Fig. 6A). However, when we measured autophagic flux by inhibiting autophagosome-lysosome fusion with bafilomycin A1, late-passage myoblasts demonstrated lower LC3-II levels than their early-passage counterparts. Therefore, autophagic flux is decreased in C2C12 senescent myogenic progenitor cells (Fig. 6B).

On the other hand, C2C12 myoblats-myotubes transition was accompanied by increased autophagy. We found that the protein levels of Beclin-1, LC3-I and LC3-II suffered a significant progressive increase during the differentiation process of both early- and late-passage C2C12 cells, following a similar pattern. However, late-passage Dif7d C2C12 cells presented lower levels of LC3-I and LC3-II than early-passage Dif7d C2C12 cells. Despite this, while differentiation of early-passage C2C12 myoblasts into myotubes produced a decrease in SQSTM1/p62 protein content supporting increased autophagy, no changes in SQSTM1/p62 levels were observed along the differentiation of late-passage C2C12 myoblasts (Fig. 6A). The accumulation of LC3-II after the treatment with bafilomycin A1 and along the differentiation process of early- and late-passage C2C12 cells confirms the activation of autophagy upon cell differentiation. Surprisingly, although late-passage myoblasts presented lower autophagic degradation rates than their early-passage counterparts, late-passage differentiating cells displayed a higher autophagic response than early-passage differentiating cells (Fig. 6B).

### Autophagy inhibition differentially regulate cell fate in young and senescent C2C12 cells

**Fig. 7:**
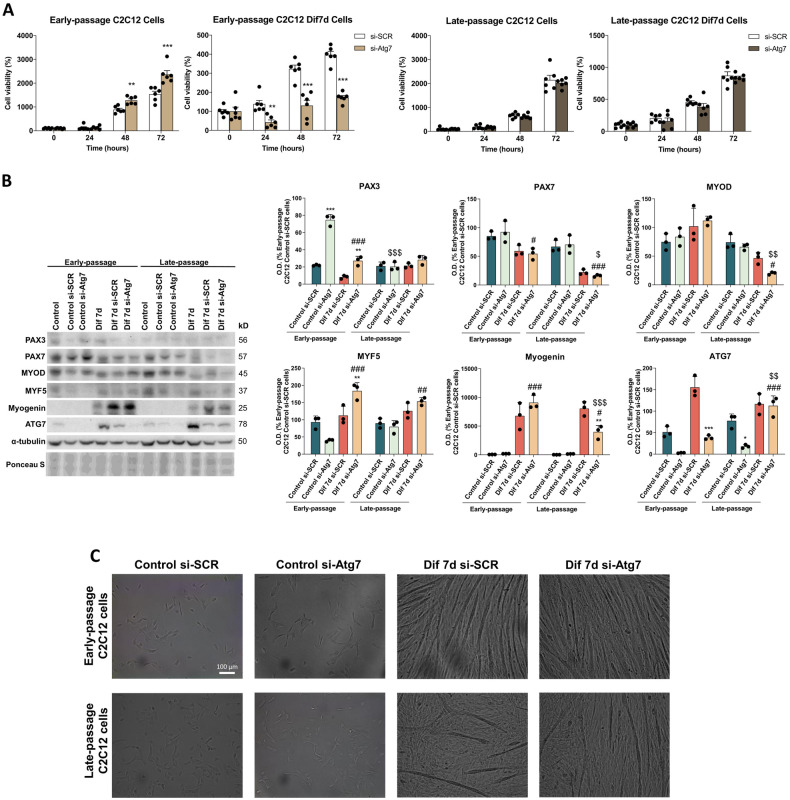
Silencing the autophagy-specific gene Atg7 differentially alters cell fate in young and senescent C2C12 cells. **A** Effect of Atg7 silencing through (si-Atg7) transfection on cell viability of Early- and Late-passage Control C2C12 and Dif7d C2C12 cells after 0-, 24-, 48- and 72 h treatment (*n* = 6). Data are mean ± SD. Statistical comparisons: *si-SCR vs. si-Atg7. The number of symbols represents the level of statistical significance: one for *P* < 0.05, two for *P* < 0.01 and three for *P* < 0.001. **B** Protein expression analysis of myogenic markers (PAX3, PAX7, MYOD, MYF5 and myogenin) and ATG7 levels in Early- and Late-passage Control C2C12 myoblasts and differentiating myotubes on day 7 (Dif7d) transfected with Atg7 (si-Atg7) or with scrambled siRNA (si-SCR) (*n* = 3). Data are mean of optical density (O.D.) ± SD expressed as percentage of Early-passage Control si-SCR C2C12 cells. Ponceau staining was used as loading control. Statistical comparisons: *si-SCR vs. si-Atg7; # Control vs. Dif7d; $ Early- vs. Late-passage. The number of symbols represents the level of statistical significance: one for *P* < 0.05, two for *P* < 0.01 and three for *P* < 0.001. **C** Phase contrast light microscopy of proliferating Early- and Late-passage Control si-SCR and si-Atg7 cells and differentiating myotubes Dif7d si-SCR and Dif7d si-Atg7 (*n* = 3). Scale bar represents 100 μm.

We next transfected early- and late-passage C2C12 cells with autophagy-related 7 (Atg7) siRNA (si-Atg7) to inhibit autophagy and explored its relevance to cell viability/proliferation and differentiation. The transfection efficiency of siRNA-mediated depletion of Atg7 was around 81% in early-passage C2C12 myoblasts and 94% in late-passage C2C12 myoblasts after 24 h and remained constant for 96 h (Supplementary Fig. 3). Atg7 silencing differently impacted early- and late-passage cells. On one way, downregulation of autophagy with si-Atg7 resulted in opposite effects on cell viability of early-passage undifferentiated and differentiated C2C12 cells, increasing the proliferation of early-passage C2C12 myoblasts, whereas decreasing the viability of early-passage C2C12 myotubes. On the other hand, the inhibition of autophagy by Atg7 silencing did not alter the viability/proliferation of both undifferentiated and differentiated late-passage C2C12 cells (Fig. 7A).

Since autophagy activity seems to be determinant for a proper differentiation pathway, we have also analyzed the impact of Atg7 silencing on the expression of myogenic markers. Early-passage Atg7-silenced myoblasts showed higher proliferation rates, higher PAX3 protein expression and no changes on the protein levels of other myogenic markers. However, all the myogenic markers remained unaltered after Atg7 silencing in late-passage C2C12 myoblasts. Myotubes differentiated from early-passage si-Atg7 myoblasts showed higher levels of PAX3 accompanied by increased MYF5 protein expression, a protein that mediates the restoration of the initial pool of satellite cells [38, 39]. Although the viability of late-passage differentiated C2C12 cells was not affected by autophagy inhibition, those C2C12 myotubes showed lower levels of MYOD and myogenin, indicating disrupted differentiation capacity. Additionally, in contrast to early-passage C2C12 myotubes, late-passage C2C12 myotubes recovered Atg7 levels after the 7days of differentiation in comparison with Dif7d scrambled siRNA (si-SCR) cells. However, this recovery was not enough to restore the myogenic differentiation potential of senescent C2C12 cells (Fig. 7B). Phase contrast images of si-Atg7 transfected cells did not evidence substantial morphological changes (Fig. 7C).

## Discussion

Sarcopenia is a large concern since muscle mass and strength are not easily recovered [40]. Sarcopenia is mainly caused by the altered cell fate of senescent satellite cells, which loss quiescence and the ability to differentiate and replace the lost myofibers [1, 41]. Pioneer studies have focused on rejuvenating strategies based on stem cell therapies to induce the differentiation of senescent myoblasts [42]. However, the cellular mechanisms that regulate myogenesis, as well as the primary mechanisms underlying the altered differentiation in senescence are poorly understood. In the present work, we uncover that senescence of C2C12 myogenic progenitor cells induces a metabolic reprogramming that impacts the differentiation process, for which both autophagy and the novel redox sensor p66Shc play important roles.

During growth and differentiation, satellite cells are exposed to different energy demands that are likely affected by aging. Overall, mitochondrial metabolism was found to directs differentiation of embryonic stem cells [43, 44]. However, several types of mesenchymal stem cells, which have lower capacity for self-renewal and proliferation than embryonic stem cells, mediates a metabolic reprogramming during differentiation by reducing oxidative phosphorylation and enhancing glycolysis [45]. In line with previous studies, we showed that C2C12 myogenic progenitor cells are metabolically active cells that change progressively during its differentiation into myotubes. Early-passage or “young” C2C12 myoblasts rely primarily on oxidative phosphorylation rather than on glycolysis, showing high maximal mitochondrial respiration which decreases after differentiation. Although early-passage myoblasts exhibit reduced mitochondrial biogenesis together with reduced OXPHOS machinery and mitochondrial mass, they are able to better respond to an energetic demand and increase the ATP production due to its higher spare mitochondrial respiratory capacity compared with differentiated cells. Previous studies have already observed that although mitochondrial complexes levels or mitochondrial mass are maintained over time, oxidative phosphorylation may vary. Brown adipose cells were found to contain large amounts of mitochondria where oxidation and phosphorylation processes are uncoupled to enhance thermogenesis [46]. Moreover, glycolytic suppression in multiple tumor cells leads to bioenergetic reprogramming toward mitochondrial respiration by increasing mitochondrial potential and without affecting mitochondrial mass [47]. Therefore, the increased oxidative phosphorylation response may be primarily due to an increase in mitochondrial quality, not quantity.

Furthermore, higher spare respiratory capacity implies that the mitochondria can operate at lower levels of respiration, reducing the electron leakage and ROS formation [48]. Indeed, our study reveals that the progressive decline in spare respiratory capacity during C2C12 myogenic differentiation is accompanied by a gradually increase of mitochondrial ROS emission rates. Curiously, ROS levels in differentiated cells do not correlate with cellular oxidative markers and total antioxidant activity. It has been widely described that ROS have an important role in cell differentiation, as they can act as intracellular signals. ROS production decreases stem cells-related genes expression and, therefore, its increase has been associated with the differentiation of several stem cell lineages [49, 50]. Therefore, the decreased oxidative phosphorylation and spare respiratory capacity displayed by C2C12 myotubes may be related to the increase in ROS generation to favor correct myogenic differentiation rather than to generate cellular damage, which ultimately makes the activation of antioxidant defense unnecessary.

Extensive evidence indicates that mitochondrial ROS generation is also related to the activation of p66Shc adaptor protein [21]. However, we found here that the progressive increase in mitochondrial ROS throughout differentiation is accompanied by reduced activation of p66Shc, evidencing that p66shc signaling pathway is not involved in the generation of mitochondrial ROS. Although p66Shc may act as an oxidoreductase, the principal site of mitochondrial ROS production is governed by the generation of superoxide anion from complex III and, particularly, from complex I [51]. Indeed, the effect of p66Shc activation on redox status and its involvement in the regulation of myogenic proliferation and differentiation is an increasingly controversial issue. Zaccagnini et al. postulated that the lack of p66Shc reduces oxidative stress and favors myogenic differentiation after limb ischemia [52], whereas other studies described that the lack of p66Shc results in evident phenotypic abnormalities and lack of terminal differentiation into myotubes [53]. Interestingly, Granatiero and colleagues found that running performance of p66Shc KO mice was similar to that observed in wild-type animals [54]. However, during downhill running, a kind of exercise associated with high levels of stress and ROS-induced damage [55], p66Shc KO animals were less fatigued [54]. Altogether these studies suggest that under maximal stress conditions, such as ischemic injury, the lack of p66Sch might be an appropriate strategy to avoid the excessive increase of ROS that would compromise myogenic differentiation. However, lack of p66Shc could be detrimental in other situations. Actually, a bifunctional role has been ascribed to p66Shc signaling. p66Shc may act as a double-edged sword, in such a way that p66Shc induces apoptosis in a pro-oxidant environment, and on the other hand, it plays a prosurvival role by modulating stem cells regulatory genes [56]. Moreover, it has been recently described a novel feature of p66Shc, which is activated to act as an antioxidant protein by maintaining upregulated other cellular antioxidant genes [57]. Increased antioxidant defense seems to be vital for the maintenance of stem cell identity and for the preservation of the regenerative properties of muscle stem cells and the myogenic differentiation potential [58, 59]. Interestingly, in our work C2C12 myoblasts also exhibited increased cellular antioxidant capacity in comparison to C2C12 myotubes, a pattern similar to that observed in the activated p66Shc adaptor protein. Therefore, the activation of the p66Shc in C2C12 myoblasts together with the observed increased antioxidant defense response may indicate the generation of the optimal environment to maintain the myogenic potential of skeletal muscle stem cells. Indeed, we identified here, for the first time, that p66Shc signaling pathway strongly impacts on myogenesis. The lack of p66Shc led to increased PAX3 levels in C2C12 myoblasts and greatly reduced myotubes viability. Notably, the induction of PAX3 was found to inhibit the myogenic differentiation of myoblasts [60, 61]. Hence, our work reveals a functional role of p66Shc protein in regulating C2C12 myoblasts fate and myogenesis.

Conversely, despite the age-related decline in the proliferative potential, late-passage C2C12 myoblasts display a higher metabolic profile than early-passage cells, relying on both mitochondrial metabolism and glycolysis, characteristic of cellular senescence [62]. Although late-passage myoblasts exhibit high mitochondrial respiration, the efficiency of oxidative phosphorylation is lower compared to early-passage cells and is accompanied by greater ROS production. Moreover, total antioxidant capacity and p-p66Shc were markedly lower, which seems to indicate that they are not able to cope with the oxidative stress experienced and, therefore, leads to increased lipid peroxidation. These data suggest that late-passage C2C12 myoblasts seem to be exposed to a sustained stress environment, leading to significant cellular damage. Although the p66Shc pathway is not activated at the levels found in young cells, this pathway is active in late-passage myoblasts and might be enhancing apoptosis rather than leading to the proliferation and preservation of prosurvival response observed in young C2C12 cells.

When differentiation is induced in senescent C2C12 cells, cell metabolism remains more similar to that exhibited by myoblasts, preserving spare mitochondrial capacity and not allowing the generation of the appropriate levels of ROS that are essential to mediate proper myogenic differentiation. As a lower coupled energy production and increased ROS production are characteristic of differentiated cells [49, 50, 63], here we further demonstrate that the metabolic remodeling and the oxidative environment of late-passage C2C12 cells influenced by the altered p66Shc signaling is associated with reduced myogenic differentiation capacity. Interestingly, we found that p66Shc depletion not only promoted PAX3, but also reduced PAX7 protein levels, a profile associated with enhanced ability of long-term proliferation and lower differentiation capacity [35]. More importantly, increased PAX7 and MYOD protein levels were observed in late-passage si-Shc1 myotubes, which are indicative of asynchronously proliferating conditions [36]. It has been widely described that the deletion of p66Shc protects from age related-diseases and extends lifespan [24, 25]. Taken together, our findings seem to indicate that the lack of p66Shc-mediated responses might lead the senescent slow-dividing myoblasts to acquire a long-term self-renewal profile, that could improve the skeletal muscle homeostasis throughout aging. However, the silencing of this pathway is harmful for the differentiation capacity of late-passage C2C12 cells because seems to induce an asynchronous proliferation. A similar situation has been described during the culture of primary satellite cells that were subjected to differentiation for extended periods, where a number of myogenin-negative cells were found [36]. Therefore, the inhibition of p66Shc impairs the differentiation capacity of senescent C2C12 cells and, as a result, reduces the fewer regenerative properties.

Autophagy has also been involved in the regulation of stem cell fate determination and differentiation [64, 65], probably due to the metabolic switch experienced during differentiation of stem cells. A highly metabolic profile, as described here in C2C12 myoblasts, is associated with an anabolic state to sustain proliferation [66]. Moreover, this is accompanied by a reduced autophagy flux in C2C12 myoblasts in comparison to C2C12 myotubes, as also occurs upon differentiation of other types of stem cells [67]. In fact, autophagy was also found to be activated after muscle injury to promote mitochondrial maintenance and contribute to muscle regeneration [68]. Autophagy may represent a critical mechanism to ensure cell survival under the metabolic switch. This finding suggests that autophagy takes part in the metabolic reprograming described upon C2C12 differentiation by contributing to the maintenance of cellular homeostasis and avoiding cell death.

Recent findings have described the age-related decline in autophagy activity as a driver of stem cell functional decline [69], since autophagy appears to provide nutrients necessary to meet bioenergetic demands during transition from quiescence to activation [12]. Interestingly, we found the previously described reduced autophagic response, but also reduced SQSTM1/p62 expression in late-passage myoblasts. SQSTM1/p62 is a multifunctional protein that is commonly used as a maker of autophagic flux. However, SQSTM1/p62 appear to play a different role in senescent myogenic progenitors. Loss of SQSTM1/p62 reported a failure shift from glycolysis to oxidative phosphorylation, a mild reduction in mitochondrial efficiency, delayed removal of dysfunctional mitochondria and increased ROS levels [70, 71], which has been associated to accelerated aging and reduced lifespan [72]. Therefore, the loss of SQSTM1/p62 expression accompanied by the excessive oxidative damage and defective oxidant scavenging activity, together with the reduced activity of autophagy in late-passage C2C12 myoblasts seem to be determinant for the impaired proliferation capacity. Interestingly, autophagic flux increases in late-passage cells upon differentiation. An exacerbated and delayed autophagic response was found to reinforce the senescent phenotype since favors the recycling of proteins into senescence-associated secretory phenotype factors [73, 74]. In fact, its inhibition in mesenchymal stem cells and in myoblasts with toxic stress-induced senescence diminishes the senescent state [74, 75]. Probably, the altered autophagic response turn the differentiated senescent C2C12 cells into an aged state with impaired myogenic capacity.

Our study also reveals that the inhibition of macroautophagy machinery alters cell fate, favoring early-passage C2C12 myoblasts proliferation and decreasing the proliferation of differentiated C2C12 cells. Moreover, si-Atg7 transfected C2C12 myoblasts showed increased viability and PAX3 levels, which promotes stem cell expansion and inhibits myogenic differentiation [60, 61, 76]. Accordingly, differentiated C2C12 cells exhibited decreased cell viability and increased MYF5 expression that is involved in the restoration of the initial pool of satellite cells [38, 39]. Therefore, a decreased autophagic response favors the renewal of C2C12 progenitor cells but is detrimental for the differentiation capacity and their proliferation. Thus, autophagy is key to cell fate determination and to upregulate C2C12 myogenesis.

On the other hand, while silencing autophagy resulted in reduced viability during early differentiation of young myoblasts, it had no effect on senescent counterparts. Interestingly, PAX3 levels were increased in early-passage Atg7-silenced myoblasts, whereas they remained unaltered in senescent cells, which seems to indicate that the lack of Atg7-dependent autophagy does not affect the maintenance of aged muscle progenitor cells. This could be because autophagic response is already diminished in late-passage C2C12 myogenic progenitor cells and, therefore, their self-renewal capacity is already impaired. Furthermore, autophagy inhibition led to the reduction of MYOD and myogenin, which indicate that the lack of autophagy impairs the differentiation capacity of late-passage cells, thereby retaining cells in an earlier differentiated state. Overall, these data indicate that the lack of autophagic response impairs the myogenic capacity of late-passage C2C12 cells, even more than the alteration caused by cellular senescence itself. Thus, maintaining autophagy at steady and regulated levels is determinant for C2C12 differentiation, particularly in aged cells which are more vulnerable than their younger counterparts.

In summary, the present work demonstrates that the p66Shc signaling pathway is essential for the myogenic commitment and the differentiation of C2C12 cells. Moreover, our study also supports that autophagy is critical for the metabolic switch exhibited during the differentiation of C2C12 myoblasts, validating how its regulation determines cell fate. In addition, this work suggests the regulation of p66Shc signaling and autophagy as tools for the development of new muscle stem cells therapies, ensuring a correct muscle stem cells maintenance and viability.

## Materials and Methods

### Reagents

High-glucose Duldecco’s modified Eagle’s medium (DEMEM) (D5648, Sigma-Aldrich, MO, USA); Fetal Bovine Serum (FBS) (102700-106, Gibco, MA, USA); Sodium bicarbonate (S5761, Sigma-Aldrich); Antibiotic/antimycotic solution (A5955, Sigma-Aldrich); Horse Serum (16050122, Gibco); Trypan Blue dye (T8154, Sigma-Aldrich); 2’,7’-diclorofluorescin diacetate with a thiol-reactive chloromethyl group (CM-H2DCFDA, C6827, Invitrogen, CA, USA); HBSS (14025092, Gibco); Hydrogen peroxide (H2O2) (Sigma Aldrich; 216763); MitoSOXTM Red fluorescent probe (M36008, Invitrogen); Hoechst (HY-15559A, MedChem Express); Glucose (Sigma, 7021); Glutamine (Sigma; G8540); Oligomycin (Sigma; 75351); Carbonyl cyanide-p-trifluoromethoxyphenylhydrazone (FCCP) (Sigma; C2920); Rotenone (Sigma; R8875)/antimycin (Sigma; A8674); RIPA buffer (R0278, Sigma-Aldrich); Dithiothreitol, protease inhibitor cocktail (P8340, Sigma-Aldrich); Laemmli buffer (1610737, BioRad); Nonfat dry milk (170-6404, BioRad); TBS (50 mM Tris-HCl, (pH 7.5); Chemiluminescent horseradish peroxidase substrate (WBKLS0500, Millipore Corp., Darmstadt, Germany); Bafilomycin A1 (11038, Cayman, MI, USA); Atg7 siRNA (si-Atg7) (SI00900529, Qiagen; Hilden, Germany); Shc1 siRNA (si-Shc1) (1027418, Qiagen); scrambled siRNA (si-SCR) (D-001810-03-20; Dharmacon, Bucks, UK); Lipofectamine 2000 (11668-030; Invitrogen).

### Antibodies

Primary antibodies used against proteins were the following: α-tubulin (sc-23948, Santa Cruz, Texas, USA); ATG7 (8558, Cell signaling, Danvers, MA); Beclin-1 (4445, Cell signaling); CI-20 (NDUFB8) (ab110242, Abcam, Cambridge, UK); CII-30 (SDHB) (ab14714, Abcam); CIII-Core II (UQCRC2) (ab14745, Abcam); CIV-I (MTCO1) (ab14705, Abcam); CV-a (ATP5A) (ab14748, Abcam); cytochrome C (ab110252, Abcam); LC3 (PD014, Medical & Biological Laboratories Co., LDT, Japan); MYF5 (ab125078, Abcam); MYOD (sc-760, Santa Cruz); Myogenin (ab1835, Abcam); p16 lnK4a (1661, Santa Cruz Biotechnology); PAX3 (MAB2457, R&D System, MN, USA); PAX7 (ab199010, Abcam); phospho-SHC/p66 (566807, Calbiochem); PGC-1α (ab3242, Millipore); porin (MSA03) (ab14734, Abcam); SHC (610879, BD Trasduction Laboratories); SQSTM1/p62 (H00008878-M01, Abnova, Taipe, Taiwan); TFAM (PA5-29571, Thermo Fisher, USA) and TOM20 (5490, Cell Signallin).

Secondary antibodies used were the following: goat anti-rabbit IgG secondary antibody (7074 S, Cell Signaling Technology); horse anti-mouse IgG secondary antibody (7076 S, Cell Signaling Technology).

### Cell culture and differentiation

C2C12 cell line, a sub-clone from mouse C3H muscle myoblasts were purchased from American Type Culture Collection (CRL1772, VA, USA). C2C12 cells were cultured in High-glucose Duldecco’s modified Eagle’s medium (DEMEM) (D5648, Sigma-Aldrich, MO, USA) supplemented with 10% Fetal Bovine Serum (FBS) (102700-106, Gibco, MA, USA), 1.8 g/L sodium bicarbonate (S5761, Sigma-Aldrich) and 1% antibiotic/antimycotic solution (A5955, Sigma-Aldrich) at 37 °C in a humidified atmosphere of 5% CO_2_. We use a multiple population doubling model using C2C12 cells. Thus, C2C12 myoblast (Control) were maintained in early (young) and late (senescent) PD ranges by growing them in monolayer without allowing cultures to become confluent. Cells were passaged every 48 h at a 1:15 split ratio. To achieve cellular senescence, C2C12 myoblast were cultured for at least 70 PDs until proliferative potential decreased, as described in the literature [3]. Confluent (90%) early and late-passage C2C12 cells were differentiated into myotubes by the incubation with DMEM containing 2% Horse Serum (16050122, Gibco). The differentiation medium was changed every 48 h. The experiments were performed after Dif4d and Dif7d. Cell morphology of undifferentiated (control), Dif4d, Dif7d from both types of C2C12 cells, early and late-passage, was investigated by using a phase contrast light microscope (CKX 41, Olympus, Hamburg, Germany).

### Imaging and image analysis

Early and late-passage C2C12 cells were seeded at a density of 5 × 10^3^ cells/cm and subjected to the differentiation protocol. Myotube cultures were photographed with a phase contrast microscope (CKX 41, Olympus, Hamburg, Germany) and a 10x objective. For each experimental condition were collected a total of 10 images from random locations in each of the 3 wells per condition. Using the Image J software (NIH, Frederick, MD, USA), two types of images analysis were implemented: myotube length and width. To measure myotubes length it was taken in to account the myotubes alignment, presence of an elongated structure, little to no cellular debris, and no branching points. Myotubes width was measured in 4 random locations selected across the myotubes length.

### Cell growth kinetics

C2C12 cells were seeded at a density of 5 × 10^3^ cells/cm^2^. When cells reached 80% of confluence, they were counted in a TC10 Automated Cell Counter (BioRad, CA, USA) at 1:2 dilution with Trypan Blue dye (T8154, Sigma-Aldrich). PD and DT were calculated using the following equations: PD = (Log harvested − Log seeded) / Log2 and DT = (Log2 x time) / (log harvested – log seeded). PD and DT were measured over 2 months, resulting in a total of 30 passages. The experiment was performed in order to monitor growth rates and determine C2C12 cellular replicative senescence.

### Measurement of intracellular ROS

Cellular ROS generation in early- and late-passage C2C12 myoblasts and differentiated C2C12 cells was evaluated by measuring the fluorescence of 2’,7’-diclorofluorescin diacetate with a thiol-reactive chloromethyl group (CM-H_2_DCFDA, C6827, Invitrogen, CA, USA), which enables the binding to intracellular components, thereby prolonging the cellular retention of the dye. 6.5 × 10^5^ cells of each condition were washed with PBS, spin down and resuspended in 500 µL of HBSS (14025092, Gibco) containing 10 µM CM-H_2_DCFDA. Then, cells were incubated in dark at 37 °C for 45 min and fluorescence was measured using the Cytoflex S flow cytometer (Beckman Coulter), with 492 and 530 nm of excitation and emission wavelengths, respectively. Cells treated with H_2_O_2_ were used as a positive control and a dye-free mixture of cells were used as a negative control.

### Evaluation of mitochondrial superoxide anion-dependent oxidative stress

Mitochondrial superoxide anion levels in early- and late-passage C2C12 myoblasts and differentiated C2C12 cells were evaluated using a MitoSOX^TM^ Red fluorescent probe (M36008, Invitrogen) that permeates live cells, selectively accumulates in mitochondria, and is rapidly oxidized by superoxide. Cells were incubated with 1.25 μM MitoSOX in HBSS Ca/Mg for 10 min in dark at 37 °C with a 5% CO_2_ atmosphere. Then, cell nuclei were stained by incubating with 1 µg/mL Hoechst (HY-15559A, MedChem Express) in HBSS, for 10 min at 37 °C in the dark, with a 5% CO_2_ atmosphere. Afterwards, samples were examined by confocal microscopy (Leica TCS SP8 X)), images were acquired (Leica Application Suite X version 1.8.1) and analysis was performed using Image J software (NIH, Frederick, MD, USA).

### Antioxidant defense and oxidative damage studies

The antioxidant defense was analyzed in cell extracts by measuring TAA, total SOD and CAT activities. TAA was measured using the modified ABTS/H_2_O_2_/HRP method [77, 78]. To determine total SOD activity, we measured the inhibition of hematoxylin autoxidation to the colored compound hematein, according to the method developed by Martin and colleagues [79]. Catalase activity was assayed by measuring H_2_O_2_ conversion in O_2_ and H_2_O, using H_2_O_2_ as substrate according to the previously described method [80].

LPO of cell extracts was studied by the measure of malondialdehyde (MDA) and 4-hydroxynonenal (4-HNE) end-products method, based on the condensation of the chromogene 1-methyl-2-phenylindole with either MDA or 4-HNE [81]. The carbonyl content in oxidatively modified proteins was evaluated following Levine et al. method [82], with modifications described by Coto-Montes and Hardeland [83]. The protocol is based on the reaction of 2,4-dinitrophenylhydrazine with the carbonyl groups of damaged proteins.

### Seahorse stress tests

OCR and ECAR were measured using the Seahorse XFe96 analyzer (Agilent, CA, USA) and Seahorse XF Cell Mito Stress and XF Glycolysis stress Tests, according to the manufacturer’s instructions. Early- and late-passage C2C12 control, Dif4d and Dif7d cells were seeded at a density of 3 × 10^4^ cells/well of a XF 96-well culture microplate in 80 μL of growth medium and allowed to attach for 24 h. Culture medium was replaced with 175 μL of unbuffered base DMEM (D5030, Sigma) supplemented with 25 mM glucose and 4 mM glutamine for the XF Cell Mito Stress test and supplemented only with 4 mM glutamine for the XF Glycolysis stress Test at pH 7.4 adjusted with 0.1 M NaOH. Cells were incubated for 1 h at 37 °C without CO_2_. To perform the experiment, 25 μL of each compound was pre-loaded into the respective ports of each well in the XF96 sensor cartridge. For the XF Cell Mito Stress test, the final concentrations of each reagent in the XF 96-well culture microplate injected from each port were: 3 μM oligomycin (Port A), 1 μM FCCP (Port B) and 1 μM a mix of rotenone/antimycin (Port C). For the XF Glycolysis stress test, the final concentrations of each reagent were: 10 mM glucose (Port A), 3 μM oligomycin (Port B) and 50 mM 2-Deoxy-D-glucose (Port C). OCR and ECAR data were normalized to the cell protein mass, determined by suforhodamine B (S9012, Sigma-Aldrich) colorimetric assay, and were analyzed using Wave v2.3 software (Agilent).

### Western blotting

C2C12 cells were harvested, resuspended in RIPA buffer (R0278, Sigma-Aldrich) supplemented with 2 mM dithiothreitol, protease inhibitor cocktail (P8340, Sigma-Aldrich) and phosphatase inhibitor cocktail (P5726, Sigma-Aldrich) and physically ruptured by sonication. The bradford method was used to measure protein content [84]. Samples (25–50 μg of protein) were heated at 100 °C for 5 min in a Laemmli buffer (1610737, BioRad) to be separated by electrophoresis in SDS-PAGE gel at 200 V and transferred to a polyvinylidene fluoride membranes (Immobilon TM-P; Millipore Corp., MA, USA) at 350 mA. After membrane blocking with 10% (w/v) nonfat dry milk (170–6404, BioRad) in TBS (50 mM Tris-HCl, (pH 7.5) and 150 mM NaCl) for 1 h at room temperature, membranes were incubated overnight at 4 °C with the respective primary antibodies. Each antibody was previously diluted 1:1000 in blocking buffer (2% (w/v) nonfat dry milk in TBS). After three 10 min washes in TBS-T (TBS containing 0.1% Tween-20), membranes were incubated with the horseradish peroxidase-conjugated secondary antibodies diluted 1:2500 in blocking buffer (2% (w/v) nonfat dry milk in TBS) for 1 h at room temperature. After three 10 min washes in TBS-T, membranes were developed using a chemiluminescent horseradish peroxidase substrate (WBKLS0500, Millipore Corp., Darmstadt, Germany) following manufacturer’s protocol. The density of proteins bands was analyzed quantitatively with Image Studio Lite 3.1.4 software (LI-COR Biosciences, Nebraska, USA). The densitometry results were normalized to α-tubulin, which served as a loading control and was shown as percentage of early-passage C2C12 Control cells. In those wester blots performed on si-Atg7 and si-Shc1 silenced cells and in cells previously treated with bafilomycin, since α-tubulin expression was altered, ponceau staining was used as a loading control.

### Autophagic flux assessment

Autophagic flux was determined by the measure of LC3 turnover by treating cells with bafilomycin A1 (11038, Cayman, MI, USA), which blocks the fusion of autophagosomes with lysosomes inhibiting the late degradation stage of autophagy. Early- and late-passage C2C12 control cells were seeded at a density of 5 × 10^3^ cells/cm^2^ and treated 24 h later with 0.2 μM bafilomycin A1 during 24 h. After 4- and 7-days differentiation, early- and late-passage Dif4d and Dif7d cells were treated with 0.2 μM bafilomycin A1 during 24 h. LC3 and SQSTM1/p62 were detected by western blot analysis, as described above.

### Cell transfection

Early- and late-passage C2C12 cells were seeded at a density of 5 × 10^3^ cells/cm^2^ and transfected 24 h later with either Atg7 siRNA (si-Atg7) (SI00900529, Qiagen; Hilden, Germany), Shc1 siRNA (si-Shc1) (1027418, Qiagen) or with a scrambled siRNA (si-SCR) (D-001810-03-20; Dharmacon, Bucks, UK), using lipofectamine 2000 (Invitrogen) and following the manufacturers’ instructions. Cells were cultured in culture medium from 1 to 4 days to analyze knockdown efficiency by western blot analysis against the target protein. To determine whether Atg7 and Shc1 silencing affects the differentiation capacity, early- and late-passage C2C12 control cells were subjected to the 7-day-differentiation protocol 24 h post-transfection. Cell morphology of young and senescent C2C12 control and Dif7d transfected cells was investigated by using a phase contrast light microscope (CKX 41, Olympus, Hamburg, Germany).

### Cell viability/proliferation assay

C2C12 control and Dif7d (early- and late-passage) were seeded at a density of 5 × 10^3^ cells/cm^2^ in 96-well plates, allowed to attach for 24 h and then transfected with either si-Atg7, si-Shc1 or with si-SCR. Cell density was measured after 0, 24, 48, and 72 h of transfection by the suforhodamine B colorimetric assay described previously [85], based on the optical density measurement of dye bound to cellular proteins.

### Statistical analysis

The statistical software package SPSS 20.0.0 (SPSS Inc., IL, USA) and GraphPad Prism 6.0 (GraphPad Software, Inc. CA, USA) for Macintosh were used for all statistical analyses and building graphs. Data are mean values ± standard deviation of the mean (SD) calculated from at least three separate experiments. The normality of the data was studied using the Kolmogorov-Smirnov test. Multiple comparisons were determined by bidirectional analysis of variance (ANOVA) to study the effects of senescence and myogenic differentiation states, followed by Bonferroni post-hoc test. Differences were considered statistically significant with *P* < 0.05.

### Supplementary information

Supplementary information

## Data Availability

All data needed to evaluate the conclusions in the article are present in the paper and/or the Supplementary Materials. Additional data related to this article may be requested from the authors.
