# Supplementary material for: p66Shc signaling and autophagy impact on C2C12 myoblast differentiation during senescence
Source: Cell Death Dis. 2024 Mar 8;15(3):200. doi: 10.1038/s41419-024-06582-0 (PMC10923948; doi:10.1038/s41419-024-06582-0)

SUPPLEMENTARY INFORMATION

p66Shc signaling and autophagy impact on C2C12 myoblast differentiation during senescence

Yaiza Potes, Juan C. Bermejo-Millo, Catarina Mendes, José Pedro Castelão-Baptista, Andrea Díaz-Luis, Zulema Pérez-Martínez, Juan J. Solano, Vilma A. Sardao, Paulo J. Oliveira, Beatriz Caballero, Ana Coto-Montes, Ignacio Vega-Naredo

SUPPLEMENTARY FIGURES

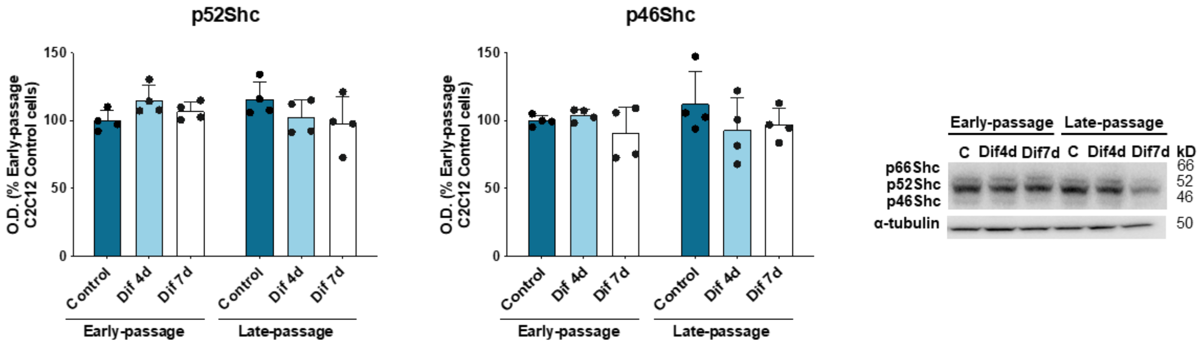

Supplementary Figure 1. Protein expression analysis of the Shc-transforming protein 1 p52Shc and p46Shc (*n* = 4). Data are mean of optical density (O.D.) ± SD expressed as percentage of Early-passage C2C12 control cells. α-tubulin was used as loading control. Statistical comparisons: *Control vs. Differentiation; # Dif4d vs. Dif7d; $ Early-passage vs. Late-passage. The number of symbols represents the level of statistical significance: one for $P < 0.05$, two for $P < 0.01$ and three for $P < 0.001$

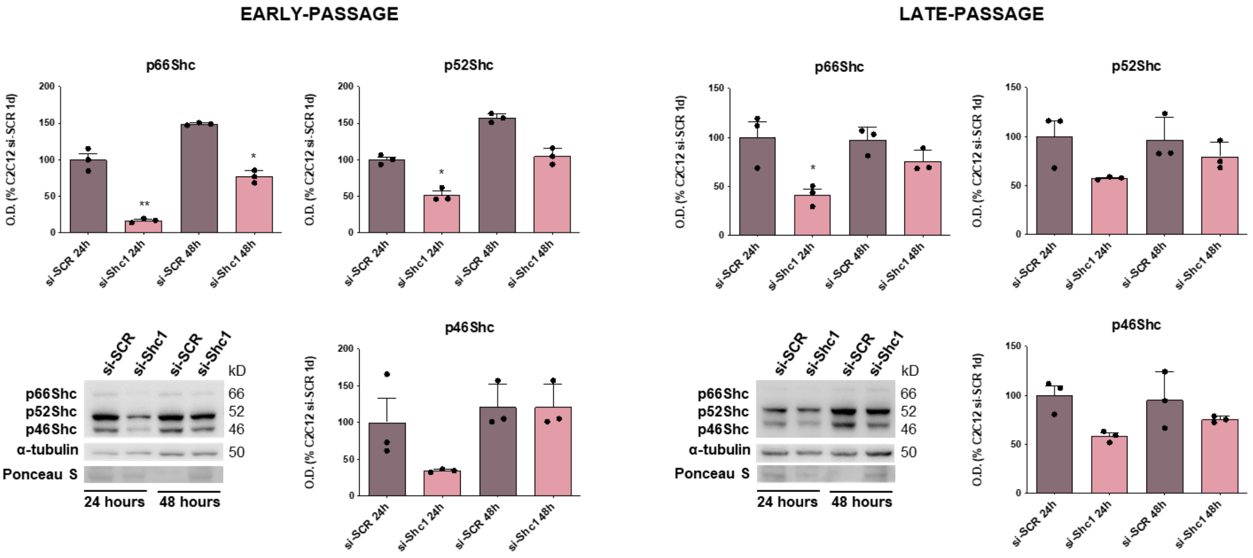

Supplementary Figure 2. Representative inmunoblot for detecting p46Shc, p52Shc, p66Shc and p-p66Shc expression overtime after transfection of Early- and Late-passage C2C12 cells with either Shc1 siRNA oligonucleotide (si-Shc1) or with a scrambled siRNA (si-SCR) (n = 3). *si-SCR vs. si-Shc1. The number of symbols represents the level of statistical significance: one for $P < 0.05$, two for $P < 0.01$ and three for $P < 0.001$

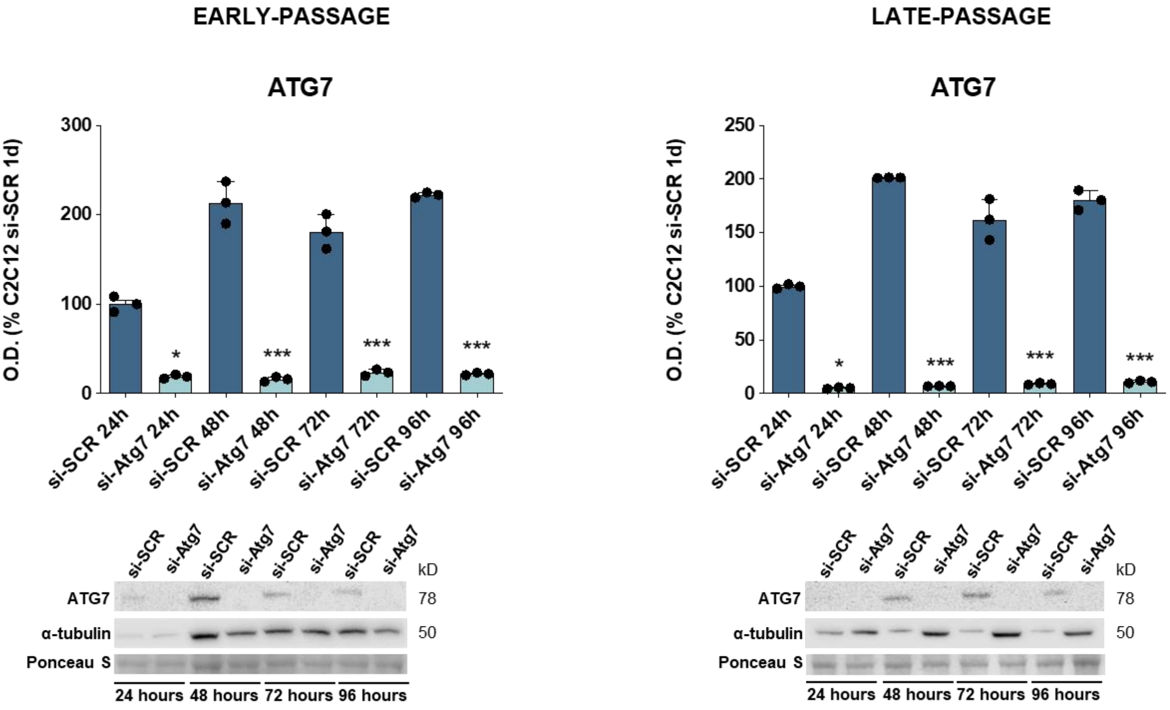

Supplementary Figure 3. Representative inmunoblot for detecting Atg7 expression overtime after transfection of Early- and Late-passage C2C12 cells with either Atg7 siRNA oligonucleotide (si-Atg7) or with a scrambled siRNA (si-SCR) (*n* = 3). *si-SCR vs. si-Atg7. The number of symbols represents the level of statistical significance: one for $P < 0.05$, two for $P < 0.01$ and three for $P < 0.001$

UNCROPPED BLOTTING IMAGES

Figure 1A

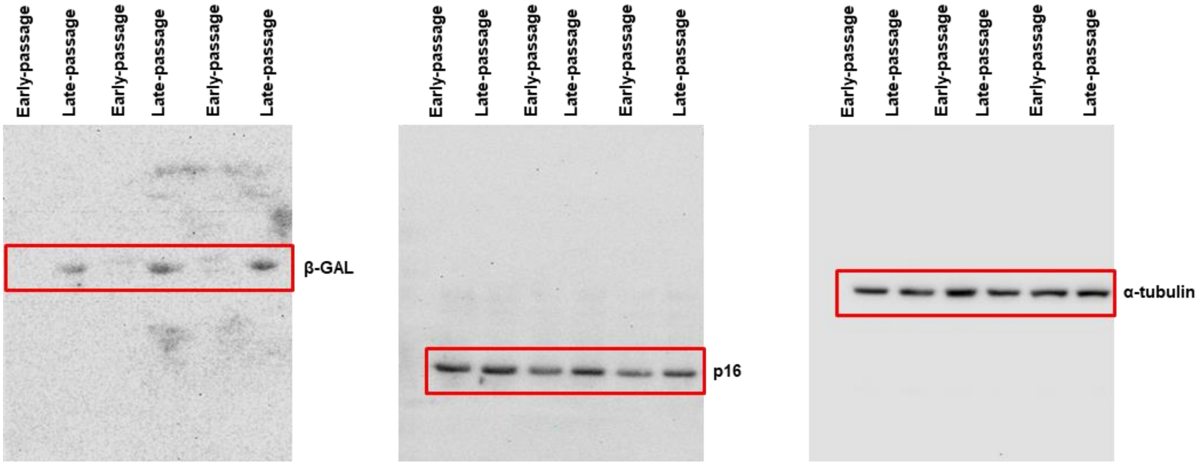
Early-passage
Late-passage
Early-passage
Late-passage
Early-passage
Late-passage
β-GAL
Early-passage
Late-passage
Early-passage
Late-passage
Early-passage
Late-passage
p16
Early-passage
Late-passage
Early-passage
Late-passage
Early-passage
Late-passage
α-tubulin

Figure 1B

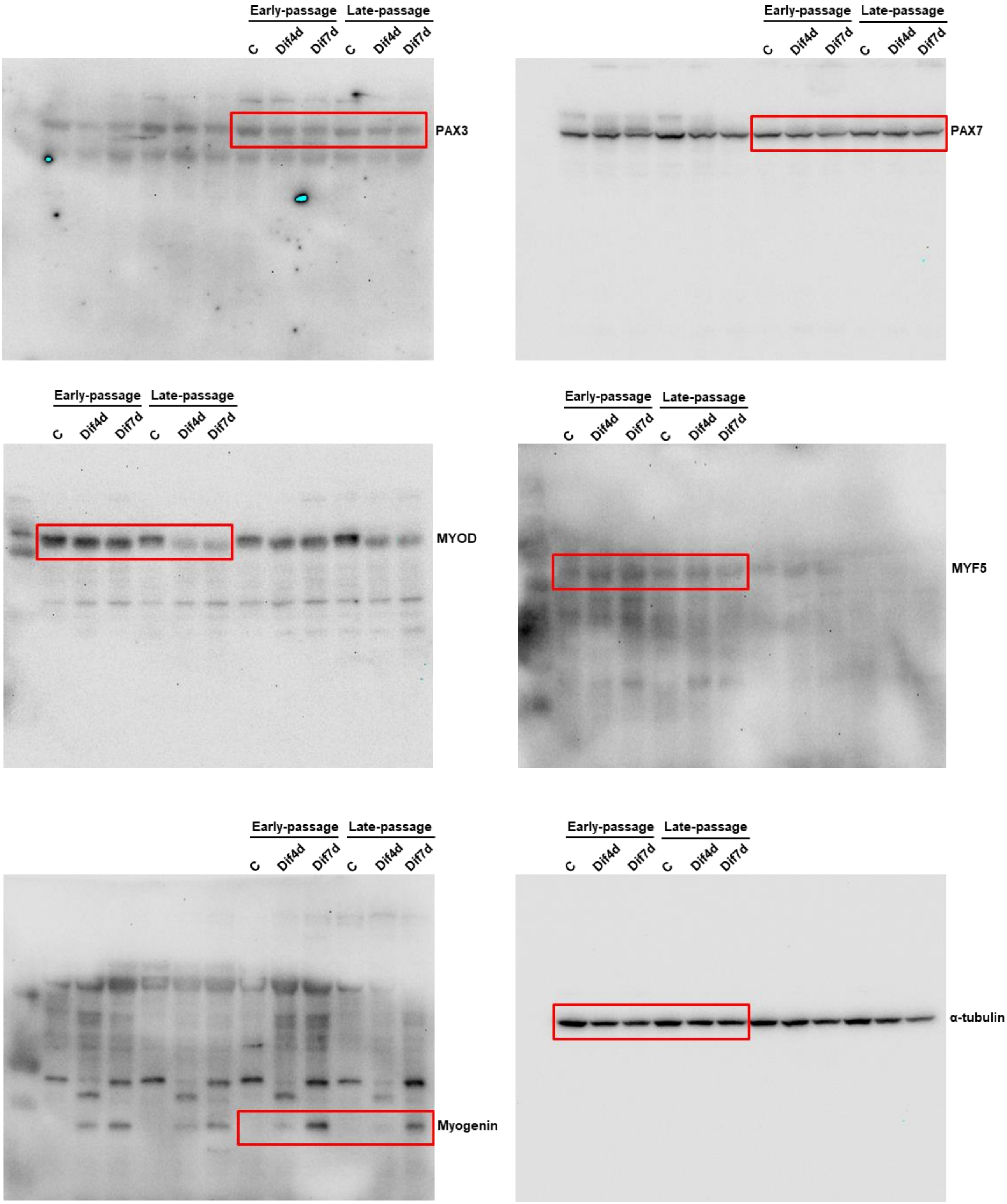
Early-passage
Late-passage
C
Dif4d
Dif7d
C
Dif4d
Dif7d
PAX3
PAX7
MYOD
MYF5
Myogenin
α-tubulin

Figure 2A

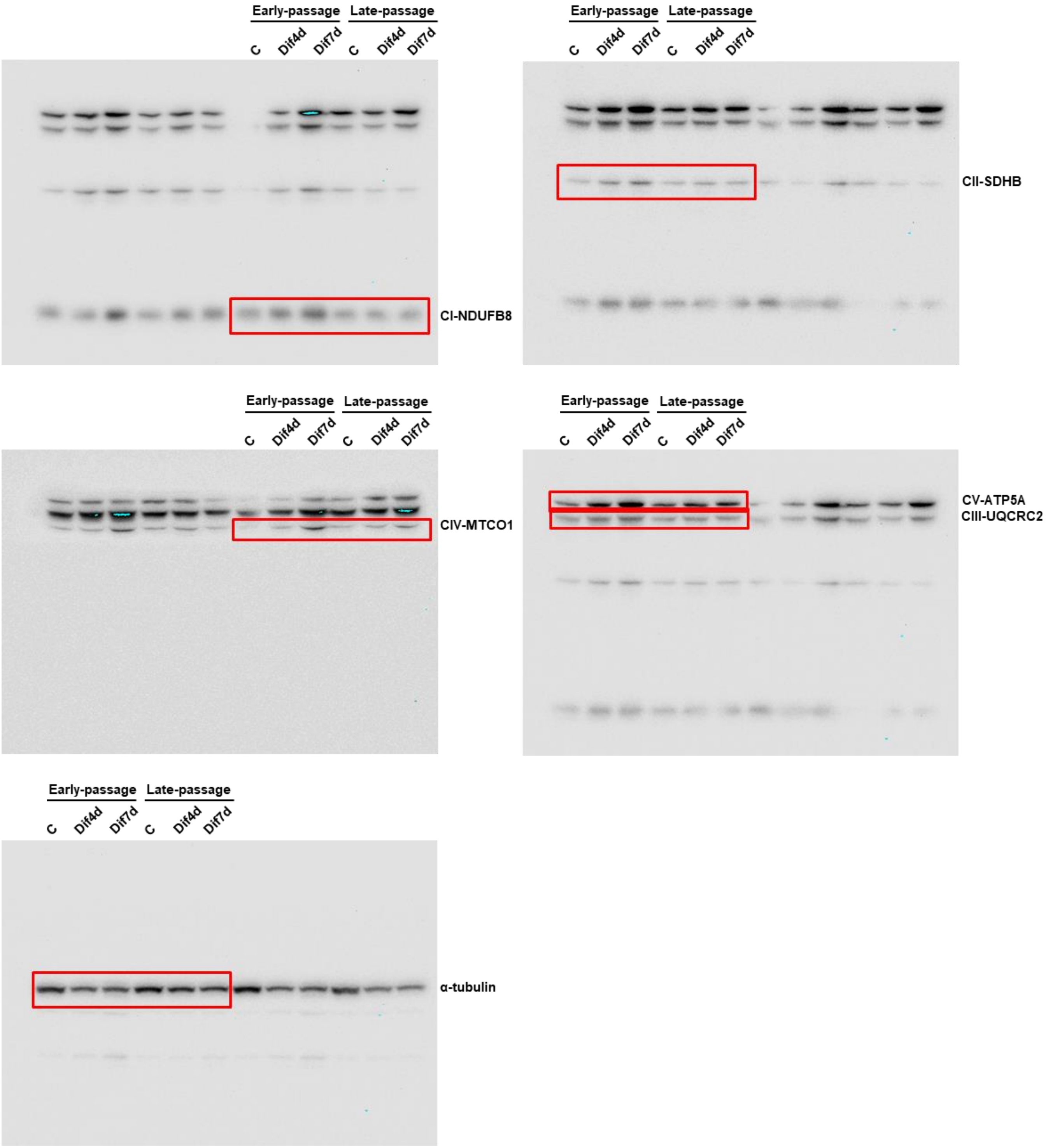
Early-passage
Late-passage
C
Dif4d
Dif7d
C
Dif4d
Dif7d
CI-NDUFB8
CII-SDHB
CIV-MTCO1
CV-ATP5A
CIII-UQCRC2
α-tubulin

Figure 2B

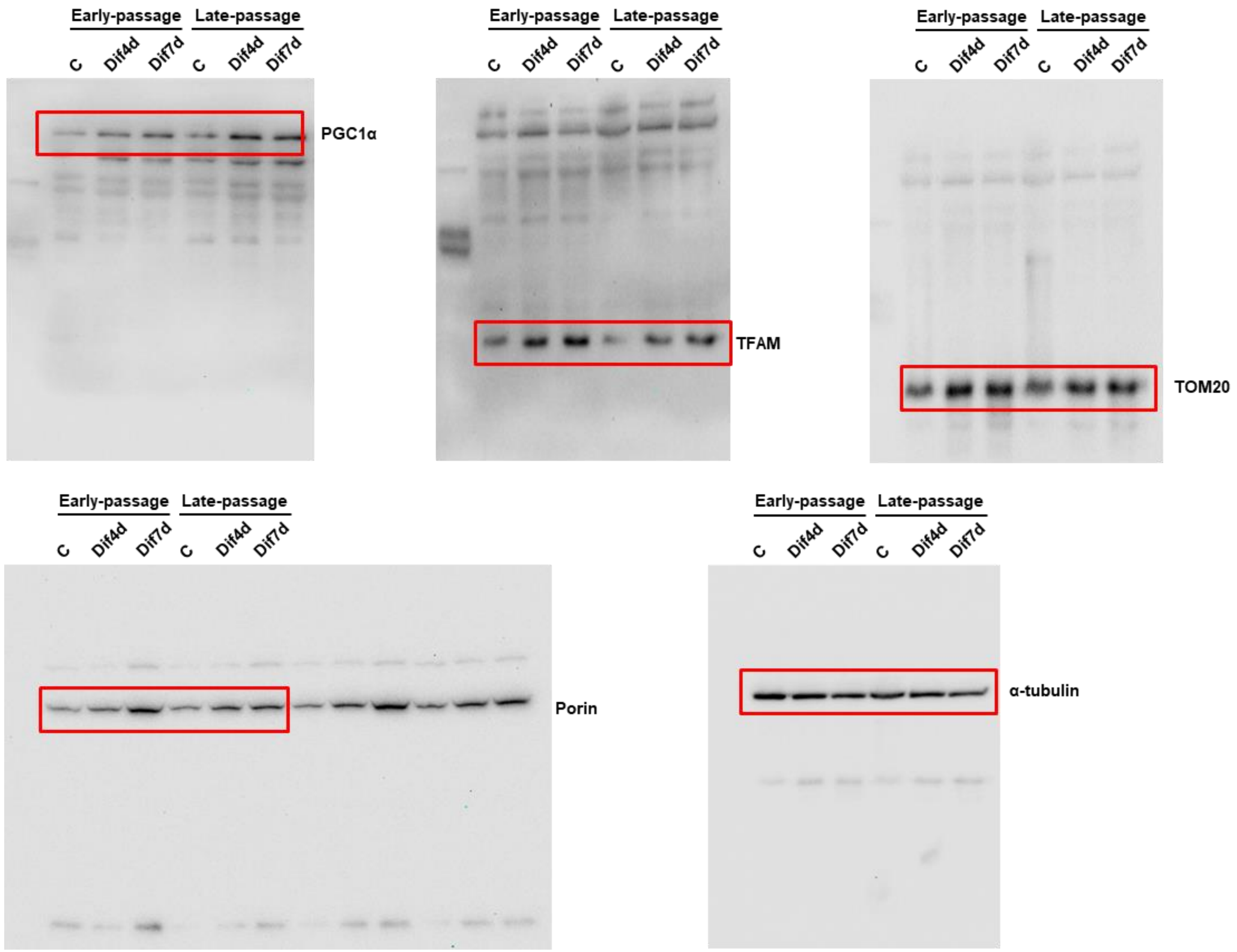
Early-passage
Late-passage
C
Dif4d
Dif7d
C
Dif4d
Dif7d
PGC1α
TFAM
TOM20
Porin
α-tubulin

Figure 4E

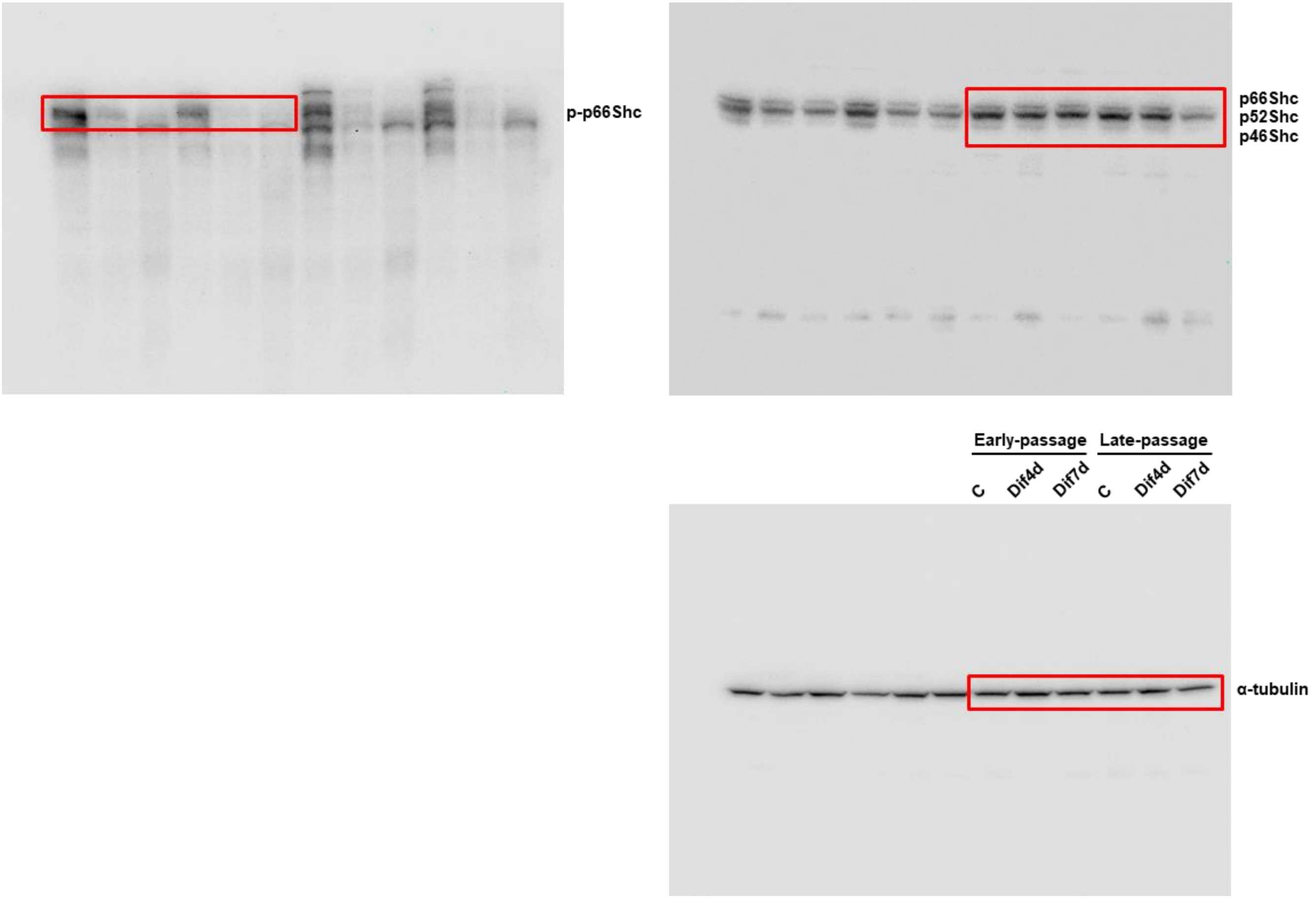

p-p66Shc
p66Shc
p52Shc
p46Shc
Early-passage
Late-passage
C
Dif4d
Dif7d
C
Dif4d
Dif7d
α-tubulin

Figure 5B

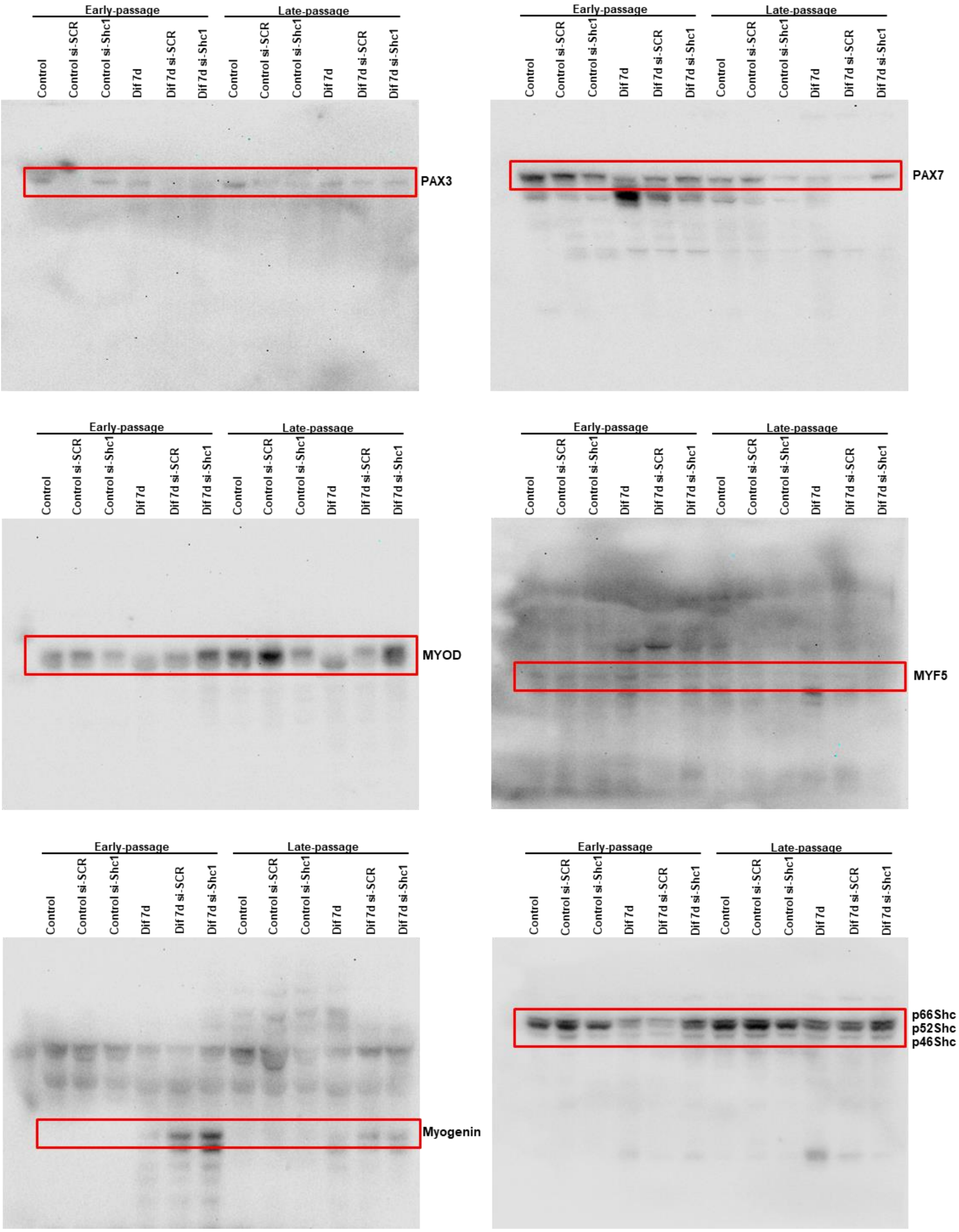
Early-passage
Late-passage
Control
Control si-SCR
Control si-Shc1
Dif 7d
Dif 7d si-SCR
Dif 7d si-Shc1
PAX3
PAX7
MYOD
MYF5
Myogenin
p66Shc
p52Shc
p46Shc

Figure 5B

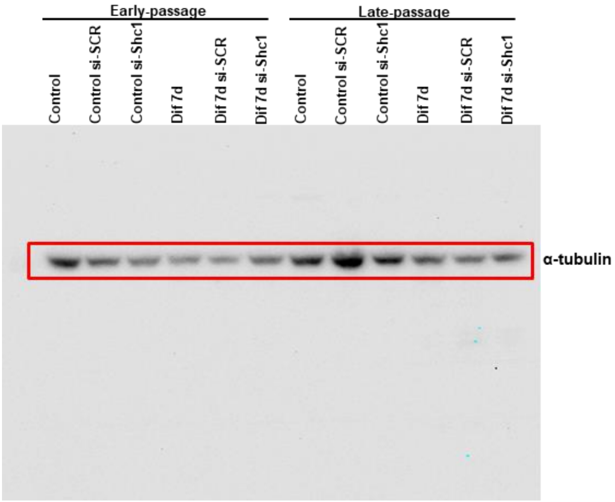
Early-passage
Late-passage
Control
Control si-SCR
Control si-Shc1
Dif 7d
Dif 7d si-SCR
Dif 7d si-Shc1
Control
Control si-SCR
Control si-Shc1
Dif 7d
Dif 7d si-SCR
Dif 7d si-Shc1
α-tubulin

Figure 6A

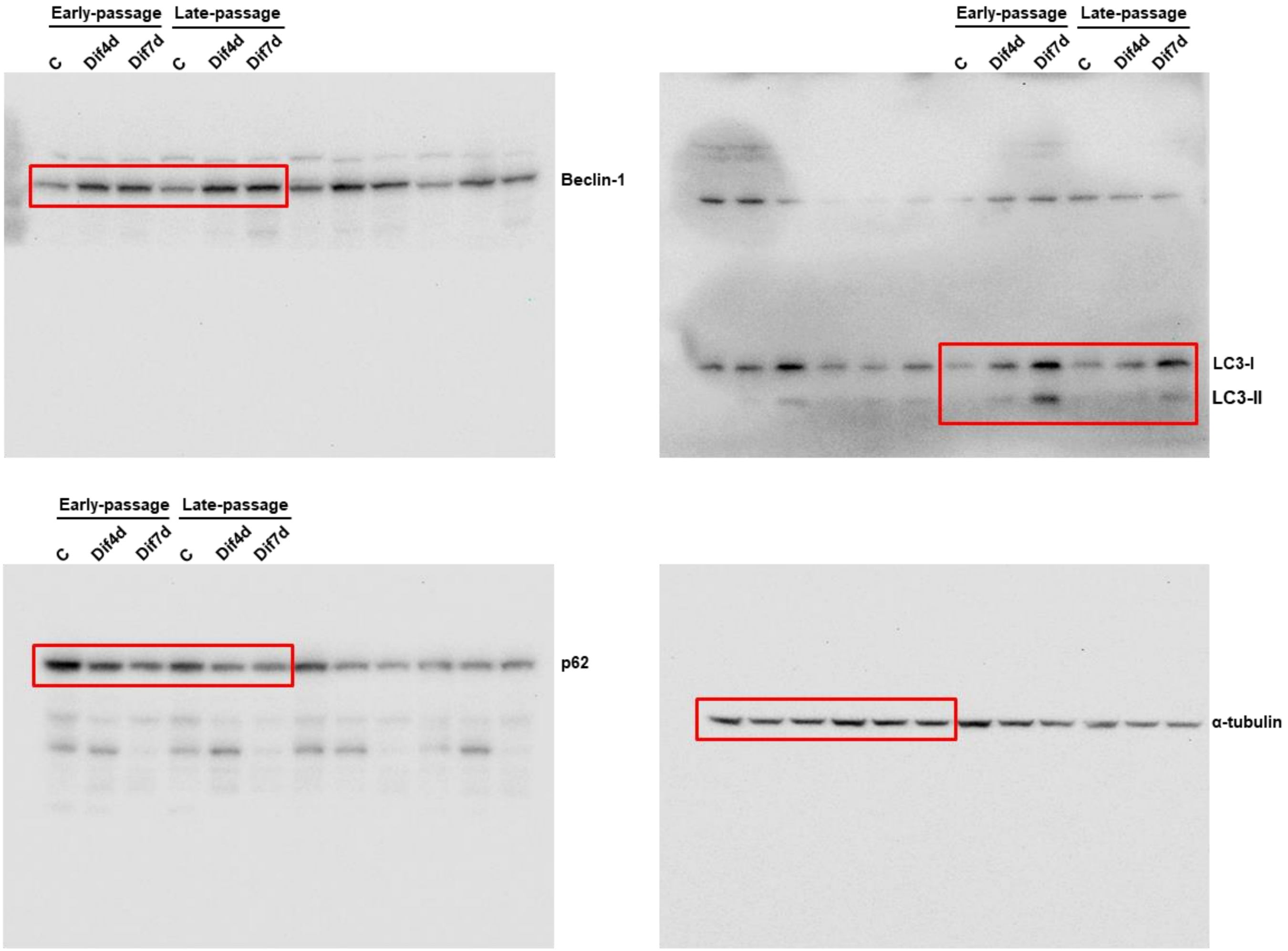

Figure 6B

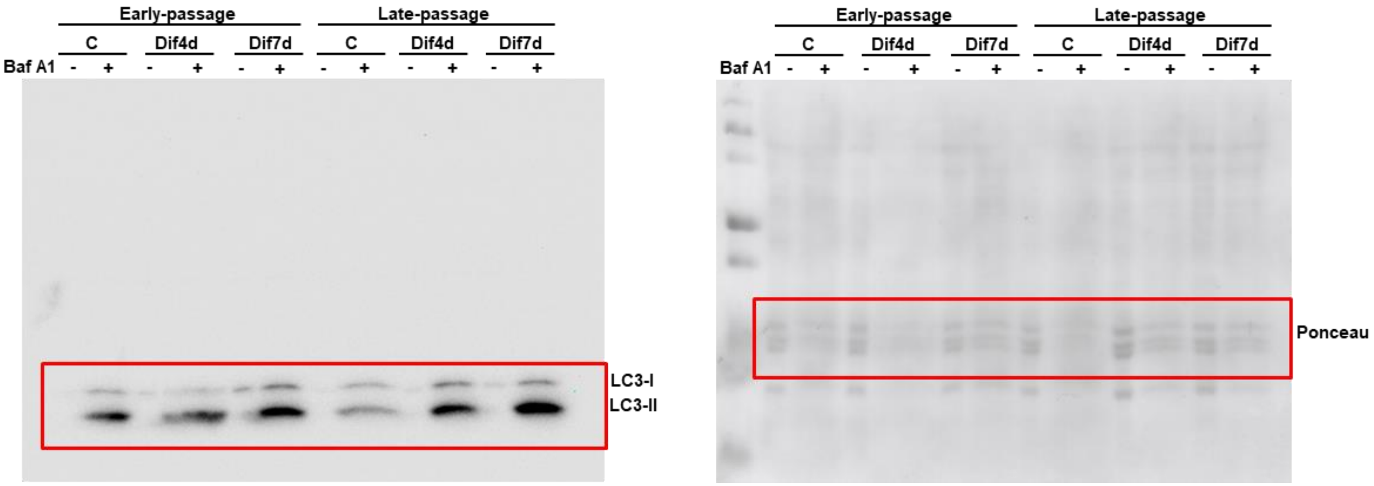

Figure 6B

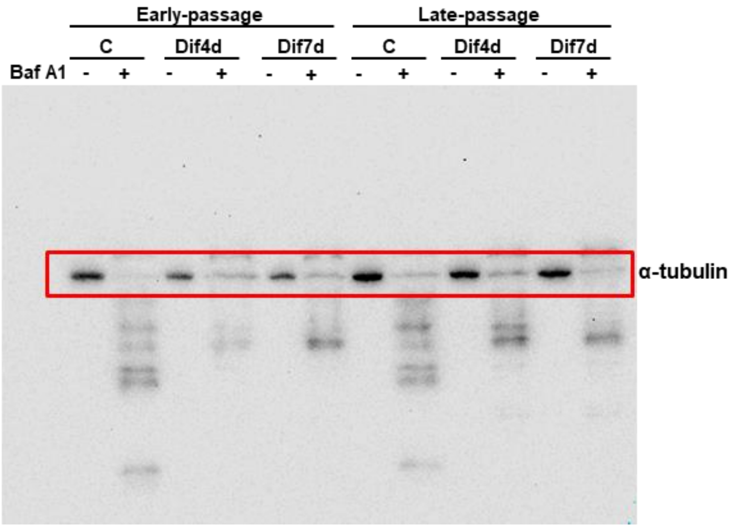
Early-passage
Late-passage
C
Dif4d
Dif7d
C
Dif4d
Dif7d
Baf A1
- + - + - + - + - + - +
α-tubulin

Figure 7B

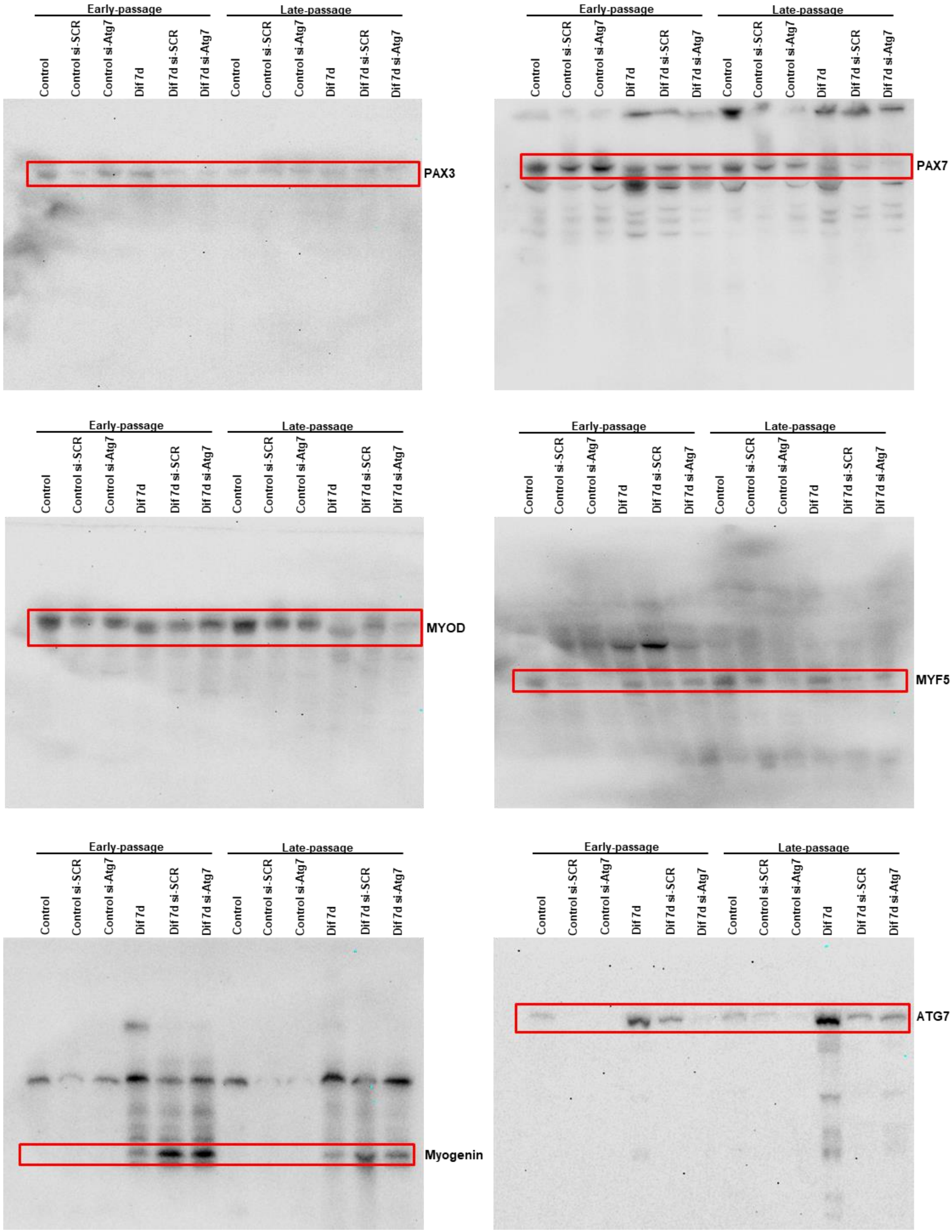
Early-passage
Late-passage
Control
Control si-SCR
Control si-Atg7
Dif 7d
Dif 7d si-SCR
Dif 7d si-Atg7
PAX3
PAX7
MYOD
MYF5
Myogenin
ATG7

Figure 7B

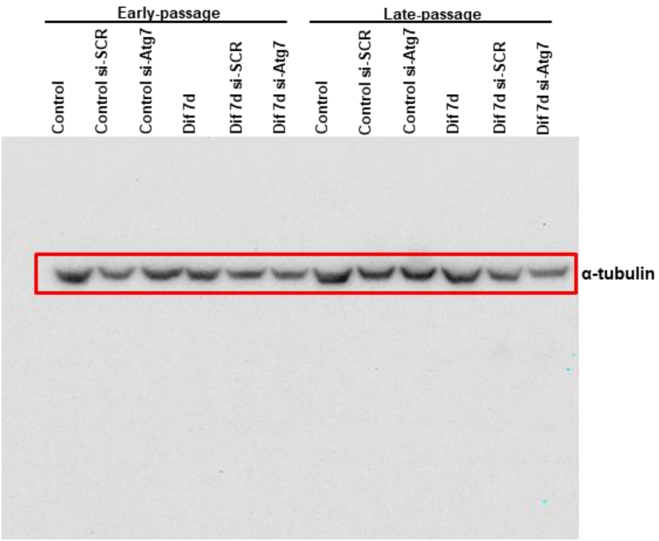
Early-passage
Late-passage
Control
Control si-SCR
Control si-Atg7
Dif 7d
Dif 7d si-SCR
Dif 7d si-Atg7
Control
Control si-SCR
Control si-Atg7
Dif 7d
Dif 7d si-SCR
Dif 7d si-Atg7
α-tubulin

Supplementary Figure 1

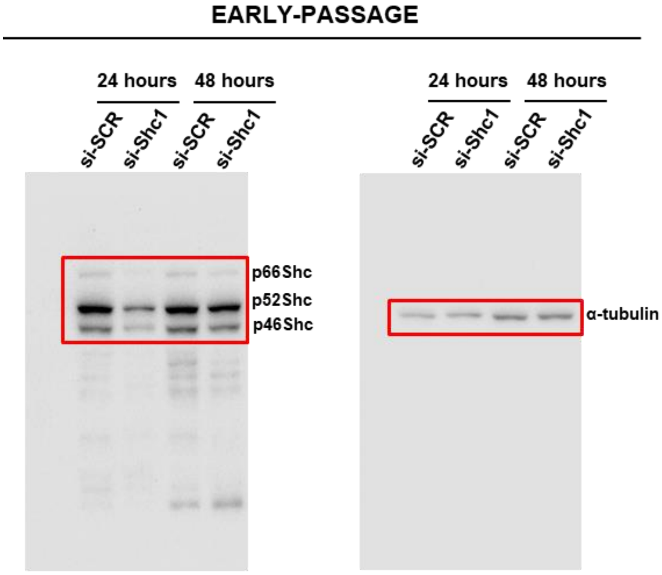

Supplementary Figure 2

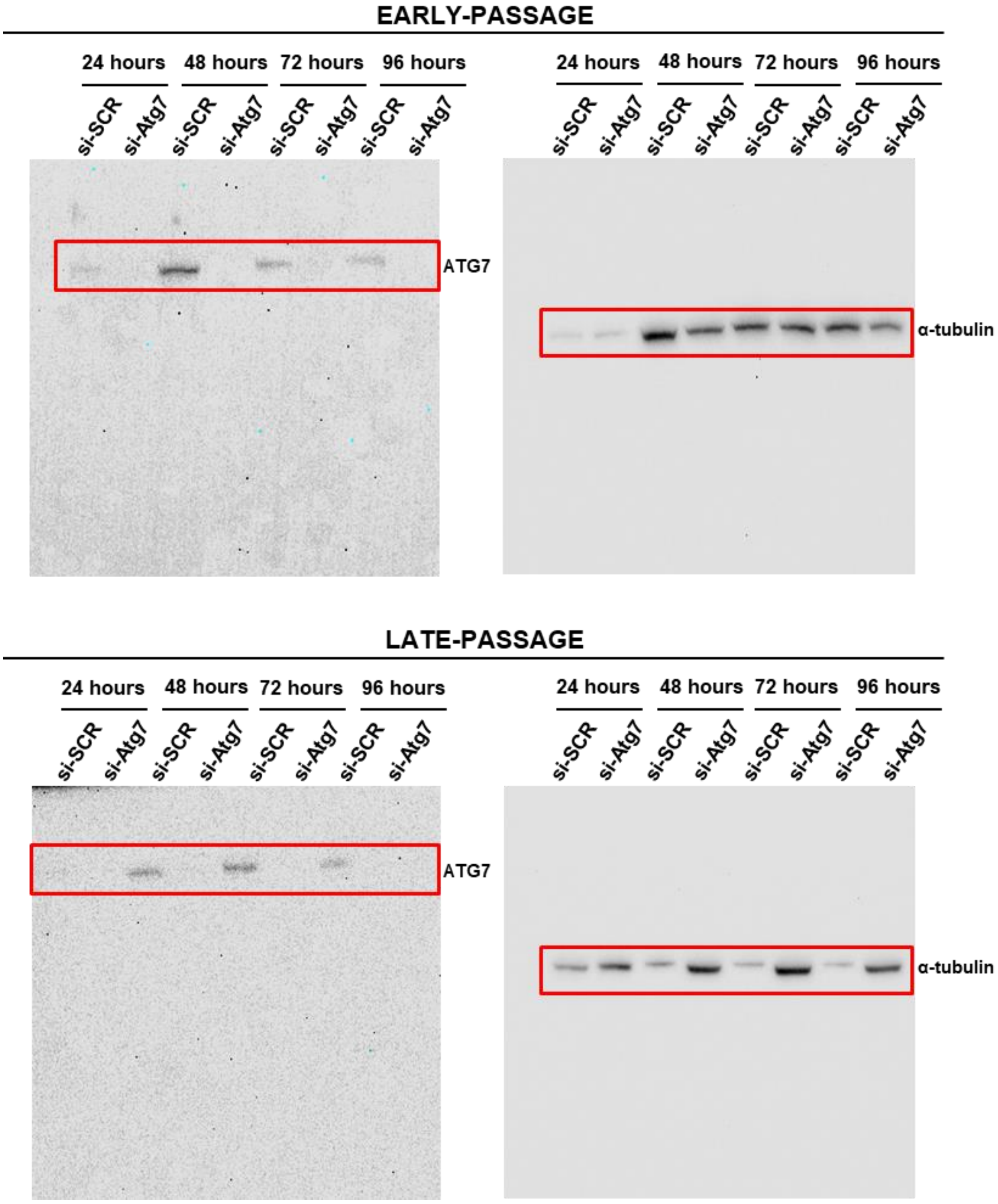

Supplement: Supplementary file 1 — Supplementary information [file 41419_2024_6582_MOESM1_ESM.pdf]
